# Chronic Cellular NAD Depletion Activates a Viral Infection‐Like Interferon Response Through Mitochondrial DNA Leakage

**DOI:** 10.1111/acel.70135

**Published:** 2025-06-16

**Authors:** Claudia C. S. Chini, Laura Colman, Eduardo Palmieri, Jessica L. Strange, Sonu Kashyap, Bing Han, Andres Benitez‐Rosendo, Gina L. Ciccio Lopez, Sara Peixoto Rabelo, Shreyartha Mukherjee, Gustavo H. de Souza, John Varga, Ralph G. Meyer, Mirella L. Meyer‐Ficca, Eduardo N. Chini

**Affiliations:** ^1^ Metabolism and Molecular Nutrition Laboratory, Kogod Center on Aging, The Glenn Foundation for Medical Research at the Mayo Clinic, Department of Anesthesiology and Perioperative Medicine Mayo Clinic College of Medicine Jacksonville Florida USA; ^2^ Department of Quantitative Health Sciences Mayo Clinic College of Medicine Jacksonville Florida USA; ^3^ Department of Internal Medicine University of Michigan Medical School Ann Arbor Michigan USA; ^4^ Department of Clinical Veterinary and Life Sciences, College of Veterinary Medicine Utah State University Logan Utah USA

## Abstract

Nicotinamide adenine dinucleotide (NAD) is a key coenzyme involved in energy metabolism, DNA repair, and cellular signaling. While the effects of acute NAD depletion have been better characterized, the consequences of chronic NAD deficiency remain unclear. Here, we investigated the impact of chronic NAD depletion in cultured cells by removing the availability of nicotinamide (NAM), a key precursor for NAD synthesis, from the culture media. In NIH3T3 fibroblasts, NAM depletion caused a dramatic drop in intracellular NAD levels within 2 days. Remarkably, the cells remained viable even after 7–14 days of NAM depletion, despite NAD^+^ levels falling to less than 10% of control conditions. This chronic NAD depletion led to distinct metabolic alterations. Mitochondrial basal respiration remained unchanged, but cells exhibited reduced spare respiratory and maximal capacities, along with significantly impaired glycolysis. Notably, NAD depletion triggered an interferon‐dependent inflammatory response, resembling viral infections. This was driven by cytosolic leakage of mitochondrial DNA (mtDNA) through voltage‐dependent anion channel 1 (VDAC1), which activated the cGAS‐STING signaling pathway. Inhibition of VDAC oligomerization with VBIT‐4, STING signaling with H‐151, or mtDNA depletion blocked the upregulation of interferon genes induced by NAM depletion. Similar interferon responses triggered by NAD depletion were observed in IMR90 human fibroblasts and HS5 stromal cells. Our findings reveal a novel link between chronic NAD deficiency, VDAC‐mediated mtDNA release to the cytoplasm, and the activation of the inflammatory response, providing new insight into how NAD decline affects cellular metabolic and inflammatory processes.

## Graphical abstract

Chronic NAD depletion in fibroblasts lowers NAD levels, increases mitochondrial mass, and triggers an interferon‐dependent inflammatory response via mtDNA leakage through VDAC1, activating the cGAS‐STING pathway. This links NAD deficiency to metabolic and inflammatory processes, with implications for diseases associated with NAD decline.

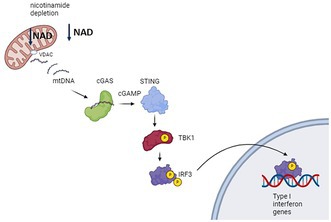

## 1 Introduction

Nicotinamide adenine dinucleotide (NAD) is a fundamental coenzyme and substrate involved in cellular energy metabolism and a variety of vital regulatory and signaling pathways (Amjad et al. 2021; Cantó et al. 2015; Nikiforov et al. 2015). Cellular NAD levels are dynamic and are regulated by the balance between its synthesis and degradation (Strømland et al. 2021). Diet plays a crucial role in providing NAD precursors necessary for its synthesis, including vitamin B3 derivatives such as nicotinamide (NAM), nicotinic acid (NA), nicotinamide mononucleotide (NMN), nicotinamide riboside (NR), and also the amino acid tryptophan (Canto 2022; Chini et al. 2023). Changes in the availability of these NAD precursors and intracellular NAD levels have been proposed to impact many key cellular functions, including metabolic pathways, mitochondrial function, DNA repair, and gene expression (Camacho‐Pereira et al. 2016; Cantó et al. 2015; Chini et al. 2021; Saville et al. 2020; Xie et al. 2020). Decreased NAD levels have been linked to a range of diseases and health conditions, including metabolic diseases, neurodegeneration, inflammatory diseases, infections, and aging (Amjad et al. 2021; Chini et al. 2023; Lautrup et al. 2024). The most severe manifestation of NAD deficiency is pellagra, a disease characterized by four “D”s, namely diarrhea, dermatitis, dementia, and death (Feuz et al. 2023).

Although it is well accepted that NAD metabolism influences cellular functions through the modulation of energy processes and the activities of NAD‐dependent enzymes, the specific signaling pathways triggered by decreased availability of NAD precursors and declines in intracellular NAD remain poorly understood. Most studies exploring the effects of NAD depletion on cellular functions have employed pharmacological inhibition of Nicotinamide phosphoribosyltransferase (NAMPT), the rate‐limiting enzyme in the NAD salvage pathway, to induce NAD depletion. However, this approach elicits acute NAD depletion and often leads to cell death (Alaee et al. 2017; Chini et al. 2014; Gehrke et al. 2014; Pajuelo et al. 2018). In contrast, during dietary vitamin B3 deficiency and other disease conditions, NAD decline is both gradual and chronic (Feuz et al. 2023; Hou et al. 2021; Katsyuba et al. 2020; Palzer et al. 2018; Tarragó et al. 2018; Zapata‐Pérez et al. 2021). To better understand the cellular mechanisms underlying the effects of chronic NAD decline, model systems that reflect long‐term NAD deficiency are necessary.

In the present study, we investigated the cellular effects of chronic NAD depletion by removing the availability of nicotinamide (NAM), a precursor required for intracellular NAD synthesis. This approach allowed us to investigate the cellular effects of chronic NAD deficiency without inhibition or activation of essential enzymes involved in NAD metabolism. Our results show that chronic, direct nicotinamide depletion in fibroblasts dramatically reduces intracellular NAD^+^ levels, triggering an interferon‐dependent inflammatory response, comparable to that seen during viral infections. This response was mediated by mitochondrial DNA (mtDNA) leakage into the cytoplasm via the VDAC channel, leading to activation of the cGAS‐STING pathway. This finding is particularly interesting since cellular NAD^+^ depletion has emerged as an ancient response to phage and viral infections in bacteria and mammalian cells (Garb et al. 2022; Osterman et al. 2024; Wang et al. 2023; Zheng et al. 2022). Our study demonstrates that the mitochondrial DNA leakage–cGAS‐STING–interferon pathway plays a key role in the cellular response to vitamin B3 and NAD deficiency. By linking NAD depletion to mitochondrial dysfunction, cGAS‐STING activation, and interferon‐mediated immune response, these findings offer new perspectives on how metabolic stress drives inflammation. This has significant implications for conditions such as infections, nutritional deficiencies, and aging.

## 2 Results

### 2.1 Nicotinamide Depletion in Fibroblasts Causes Chronic NAD ^+^ Deficiency, Inhibiting Cell Proliferation, and Inducing Metabolic Adaptations

To investigate the cellular effects of NAD deficiency, we induced chronic NAD depletion in fibroblasts, which play a crucial role in inflammation and tissue repair (Davidson et al. 2021). Cells grown in culture media such as DMEM are generally exposed to 30 μM NAM. To induce chronic NAD deficiency, cells were cultured in nicotinamide (NAM) Free media. Both NAM and NAM Free media were supplemented with dialyzed FBS. The use of dialyzed FBS was essential, as no significant NAD depletion was observed in NIH3T3 cells cultured in NAM Free media containing regular (nondialyzed) FBS (Figure S1a).

**FIGURE 1:**
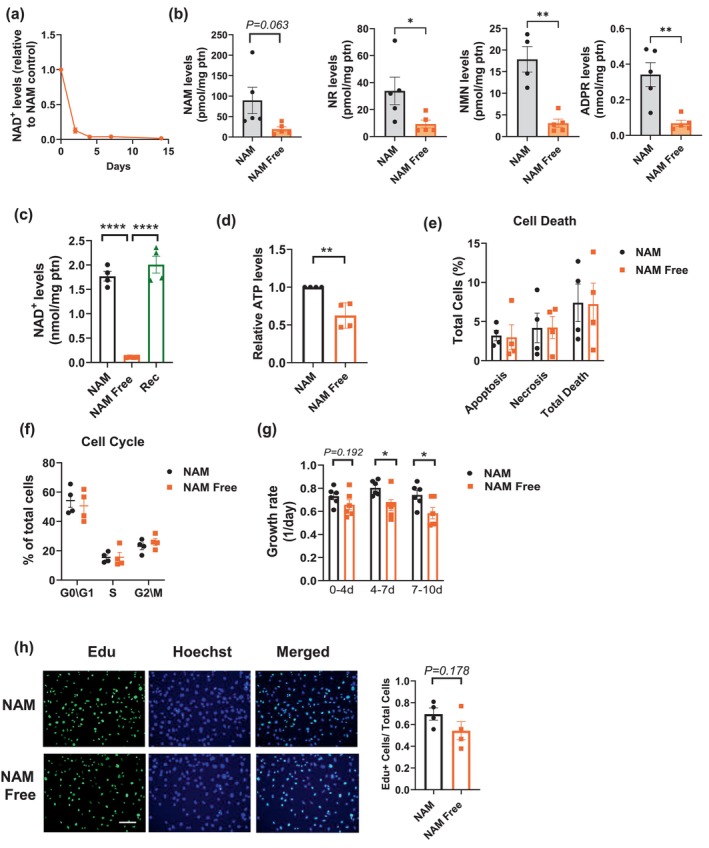
Nicotinamide depletion impairs NAD^+^ levels and cell proliferation. NIH3T3 cells were cultured in media with nicotinamide (NAM) or without NAM (NAM Free) for varying durations. (a) NAD^+^ levels in NAM Free cells expressed relative to those in NAM media (*n* = 3). (b) Levels of NAD‐related metabolites measured after 12 days of culture in NAM and NAM Free media (*n* = 5). (c) NAD^+^ levels in cells cultured for 14 days in NAM, NAM Free, or NAM Free for 9 days followed by 5 days of recovery in NAM (Recovery, Rec) (*n* = 4). (d) Relative ATP levels in cells grown in NAM and NAM Free media for 11 days (*n* = 4). (e, f) Percentage of cell death (e) and cell cycle distribution (f) in cells grown for 7 days in NAM and NAM Free media (*n* = 4). (g) Growth rates of cells cultured for different time periods in NAM and NAM Free media (*n* = 6). (h) Cell proliferation after 7 days in NAM or NAM Free media assessed by Edu incorporation assay (*n* = 4) (scale bar 41.7 μm). The data are presented as mean ± SEM, with *n* representing the number of different experiments. *p* Values were calculated using unpaired two‐sided *t*‐tests or one‐way ANOVA. *P < 0.05, **P < 0.01, ***P < 0.001, ****P < 0.0001.

NIH3T3 cells were cultured in media containing 30 μM NAM or in NAM Free media for varying durations. Surprisingly, cells remained viable for at least 4 weeks (28 days) in NAM Free media, despite NAD levels dramatically dropping to nearly undetectable levels (Figure 1). In fact, intracellular NAD^+^ levels declined to about 10% of control cells within the first 2 days and dropped to nearly undetectable levels thereafter (Figure 1a). On day 12, levels of NAD^+^, NAM, NMN, NR, and ADPR were all reduced, showing a broad decline in NAD‐related metabolites (Figure 1a,b).

Notably, when cells grown in NAM Free media for 9 days were replated in NAM media for 5 days (recovery), their NAD levels returned to those of cells continuously grown in NAM media for 14 days (Figure 1c), demonstrating the reversibility of the process. Interestingly, in cells depleted of NAM for 9 days, ATP levels were about 60% of those in cells grown in NAM media (Figure 1d). Thus, while NAD levels at this point were nearly undetectable, NAM depletion did not lead to a collapse in ATP levels. This indicates that most of the cellular NAD pool is not obligatorily coupled to ATP synthesis and that cells can partially sustain ATP levels even after substantial chronic NAD depletion.

To examine the functional consequences of chronic nicotinamide/NAD depletion, we first assessed cell growth and survival parameters in NIH3T3 cells. Despite nearly undetectable levels of NAD^+^ after 7–10 days in NAM Free conditions, there was no significant increase in apoptosis, necrosis, or evidence of cell cycle arrest (Figure 1e,f, Figure S1b). Even at 25 days in NAM Free media, cell death levels remained comparable to those in cells cultured with NAM (Figure S1c). Additionally, we did not observe significant SA‐β‐galactosidase activity, either by flow cytometry or SA‐β‐gal staining (Figure S1b,d), suggesting that these cells did not become senescent during this period. We also examined the expression of cell cycle regulatory genes, such as *p16*, *p21*, and *p53*. Although we found a small increase in *p21* mRNA expression, we did not detect a significant increase in p21 protein levels (Figure S1e,f). These results suggest that the cells were neither senescent nor cell cycle arrested during the chronic NAD depletion. However, we noted a decrease in the cell growth rate that intensified with prolonged exposure to NAM Free conditions (Figure 1g). This decrease was evident using trypan blue exclusion assay, while the EdU proliferation assay showed a trend toward reduced proliferation (Figure 1g,h). Thus, it appears that, despite reduced cell proliferation, fibroblasts acquire adaptive mechanisms to cope with very low NAD levels, allowing them to survive under chronic NAM depletion.

**FIGURE 2:**
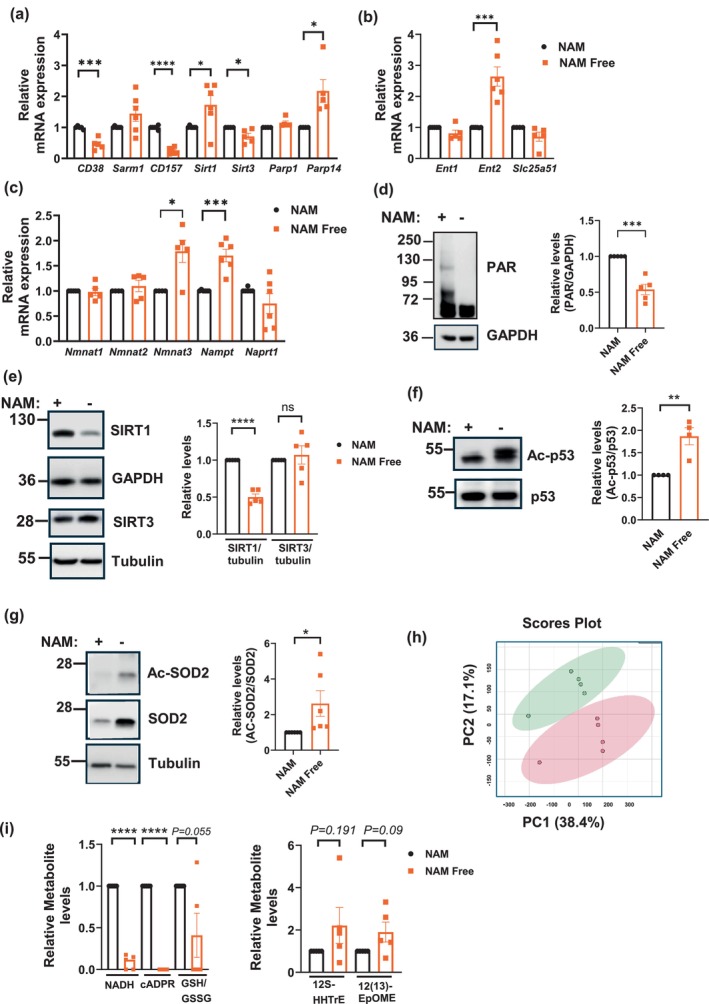
Nicotinamide depletion induces metabolic changes. NIH3T3 cells were cultured for 7–9 days in NAM or NAM Free media. (a–c) Gene expression of NAD metabolism enzymes was quantified by qPCR (*n* = 5–6). (d, e) Representative immunoblots of total PARylation (d) and SIRT1 and SIRT3 levels (e). Graphs show quantification (*n* = 5). (f, g) Representative immunoblots of Ac‐p53 and p53 (f) and Ac‐SOD2 and SOD2 (g). Graphs show quantification (*n* = 4–6). (h, i) Metabolomic analysis includes principal component analysis (h), and graphs show levels of some important metabolites (i) (*n* = 5). Data are presented as mean ± SEM, with *n* representing the number of different experiments. *p* Values were calculated using unpaired two‐sided *t*‐tests. *P < 0.05, **P < 0.01, ***P < 0.001, ****P < 0.0001.

To assess the metabolic changes induced by NAM depletion, we first examined the expression of enzymes and transporters involved in NAD metabolism. After 7–9 days of NAM depletion, we observed increased mRNA expression of the NAD synthesis enzymes NAMPT and Nicotinamide Nucleotide Adenylyltransferase 3 (NMNAT3), equilibrative nucleoside transporter 2 (ENT2), deacetylase SIRT1, and poly(ADP‐ribose) polymerase family member 14 (PARP14) (Figure 2a–c). In contrast, the expression of the NAD consuming enzymes CD38 and CD157, along with SIRT3, was reduced (Figure 2a).

At the protein level, NAM‐depleted cells showed reduced total cellular poly(ADP‐ribosyl)ation (PARylation) and SIRT1 protein levels, while NAMPT and SIRT3 showed no significant changes, although NAMPT levels trended higher (Figure 2d,e, Figure S2a). To assess the enzymatic activity of sirtuins, we measured acetylation of known substrates—p53 for SIRT1 and SOD2 for SIRT3. In line with the reduced NAD availability, the acetylation of both substrates was elevated (Figure 2f,g) indicating diminished activity of NAD‐dependent deacetylases. In the case of CD38, although changes in gene expression were observed, neither the protein (Figure S2b) nor its enzymatic activity (data not shown) were detectable in these cells. In summary, NAM/NAD depletion triggers upregulation of NAD synthesis enzymes and transporters, such as NMNAT3 (the mitochondrial NMNAT) and ENT2, while diminishing the activity of SIRT1 and SIRT3. Additionally, PARylation was markedly lower, confirming the inhibitory impact of NAM depletion on nonoxidative cellular NAD‐dependent processes.

Next, we conducted untargeted metabolomic analysis on cells cultured for 9 days in NAM and NAM Free conditions. Principal component analysis revealed a marked difference in metabolic patterns between the two conditions (Figure 2h). A heat map shows that at least 50 metabolites were significantly upregulated or downregulated in the absence of NAM (Figure S2c). As expected, metabolites related to NAD synthesis, including NADH and cyclic adenosine diphosphate ribose (cADPR) were downregulated in NAM Free conditions (Figure 2i). Additionally, a decrease in the reduced/oxidized glutathione (GSH/GSSG) ratio suggests impaired detoxification in NAM Free cells (Figure 2i). In contrast, levels of the eicosanoid derivatives 12‐(13) EpOME and 12SHHTrE, which are associated with inflammation (Lau et al. 2023), trended to increase in NAM‐depleted cells (Figure 2i). These results indicate that NAM‐depleted cells develop a severe chronic NAD deficiency that is surprisingly well tolerated, as determined by cell growth and cell cycle analysis. In addition, it suggests that NAD depletion may induce the activation of some proinflammatory pathways.

### 2.2 Nicotinamide Depletion Induces an Inflammatory Response

**FIGURE 3:**
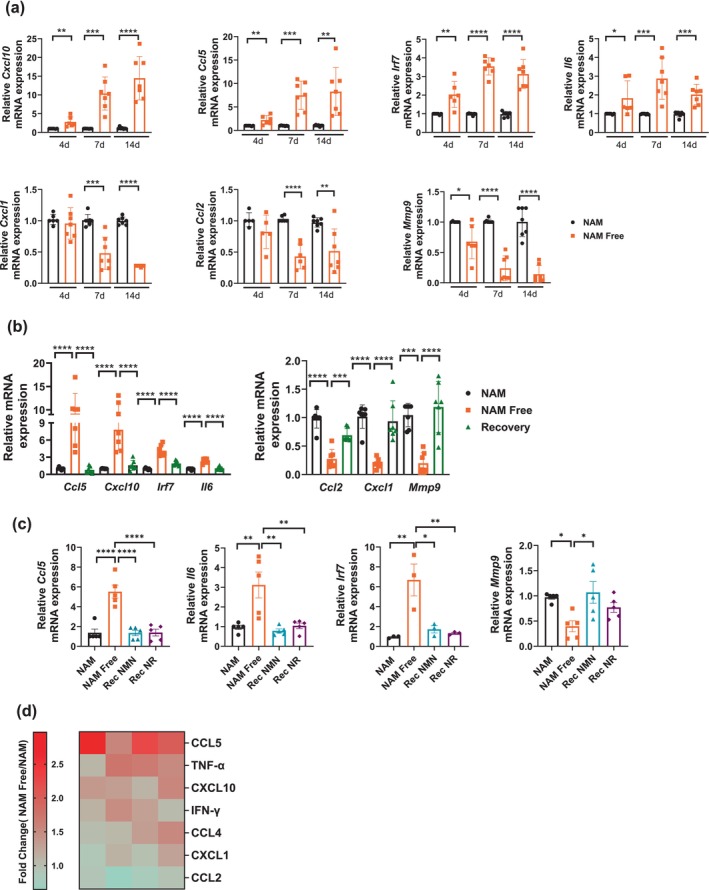
Nicotinamide depletion regulates the inflammatory response in NIH3T3 cells. (a) Cells were cultured for 4–14 days in NAM or NAM Free media (*n* = 5–7). (b) Cells were grown for 14 days in NAM, NAM Free, or NAM Free for 9 days followed by 5 days of recovery in NAM (Recovery, Rec). (a, b) Graphs show relative gene expression assessed by qPCR (*n* = 7). (c) Cells were grown for 10 days in NAM, NAM Free, or NAM Free for 7 days followed by 3 days in the presence of 30 μM nicotinamide mononucleotide (NMN) or nicotinamide riboside (NR). Graphs show relative gene expression assessed by qPCR (*n* = 3–5). (d) Heat map shows quantitative analysis of the media from cells grown in NAM and NAM Free conditions for 7–9 days. Values are expressed relative to those in NAM media (*n* = 4). Data are presented as mean ± SEM with *n* representing the number of different experiments. *p* Values were calculated using unpaired two‐sided *t*‐tests or one‐way ANOVA. *P < 0.05, **P < 0.01, ***P < 0.001, ****P < 0.0001.

The potential link between NAM/NAD depletion and cellular inflammation was further explored by investigating the inflammatory response of fibroblasts cultured for varying durations in NAM‐depleted media. In NIH3T3 cells, NAM depletion led to a time‐dependent increase in the expression of several proinflammatory cytokines, chemokines, and inflammatory genes, including *Ccl5, Cxcl10, Il6*, and *Irf7*, starting at Day 4, compared to cells grown in the presence of NAM (Figure 3a). However, NAM‐depleted cells also showed a time‐dependent decrease in the expression of genes such as *Ccl2, Cxcl1*, and *Mmp9* (Figure 3a). Importantly, all these changes were reversed when NAM‐depleted cells were switched to media containing 30 μM NAM (Figure 3b), or the NAD precursors NMN and NR (Figure 3c). This indicates that the changes in the inflammatory response are reversible and dependent on the availability of NAD precursors.

To assess changes in cytokine/chemokine secretion, we performed a quantitative analysis of the media from cells grown in both NAM and NAM Free conditions for 7–9 days. Consistent with our gene expression findings, we observed increased secretion of CCL5 and CXCL10 in NAM‐depleted cells, while CXCL1 and CCL2 either showed no change or decreased levels, measured via a Luminex assay across four independent experiments (Figure 3d). Additionally, secretion of TNF‐α, IFN‐γ, and CCL4 was also elevated in cells cultured in NAM Free media (Figure 3d). These results indicate that changes in gene expression seen in NAM Free conditions were paralleled by changes in the secretion of cytokines and chemokines.

We also investigated whether human fibroblasts responded to NAM depletion similarly to mouse fibroblasts. Consistent with our findings in NIH3T3, IMR90 human fibroblasts cultured in NAM Free media for 14 days showed a marked reduction in NAD levels, along with an elevated inflammatory response (Figure S3a,b). To determine whether this response extends to other cell lines, we conducted similar experiments using the human bone marrow stromal line HS5, which exhibits some fibroblast‐like morphology. In HS5 cells, intracellular NAD^+^ levels dramatically declined within the first 3 days and remained nearly undetectable throughout the course of the experiments (Figure S3c). Moreover, HS5 cells cultured in NAM Free media for 4–14 days exhibited an inflammatory response, with higher expression of proinflammatory genes, including *Ccl5, Cxcl10, Cxcl8, Ccl2, Irf7*, and *Infb* compared with those cultured in NAM media (Figure S3d). Similar to NIH3T3 cells, HS5 cells showed no significant changes in the expression of cell cycle arrest genes (Figure S3e), and both the decrease in NAD levels and the increase in proinflammatory gene expression were reversed when NAM Free cells were returned to media containing 30 μM NAM (Figure S3f,g). Notably, in contrast to NIH3T3 cells, we did not observe decreased expression of any inflammatory genes in HS5 cells (Figure S3d), suggesting that the phenotype induced by NAM/NAD depletion may be cell‐type specific.

### 2.3 Nicotinamide Depletion Activates a Cellular Viral Infection‐Like Interferon Response and the cGAS‐STING Pathway

**FIGURE 4:**
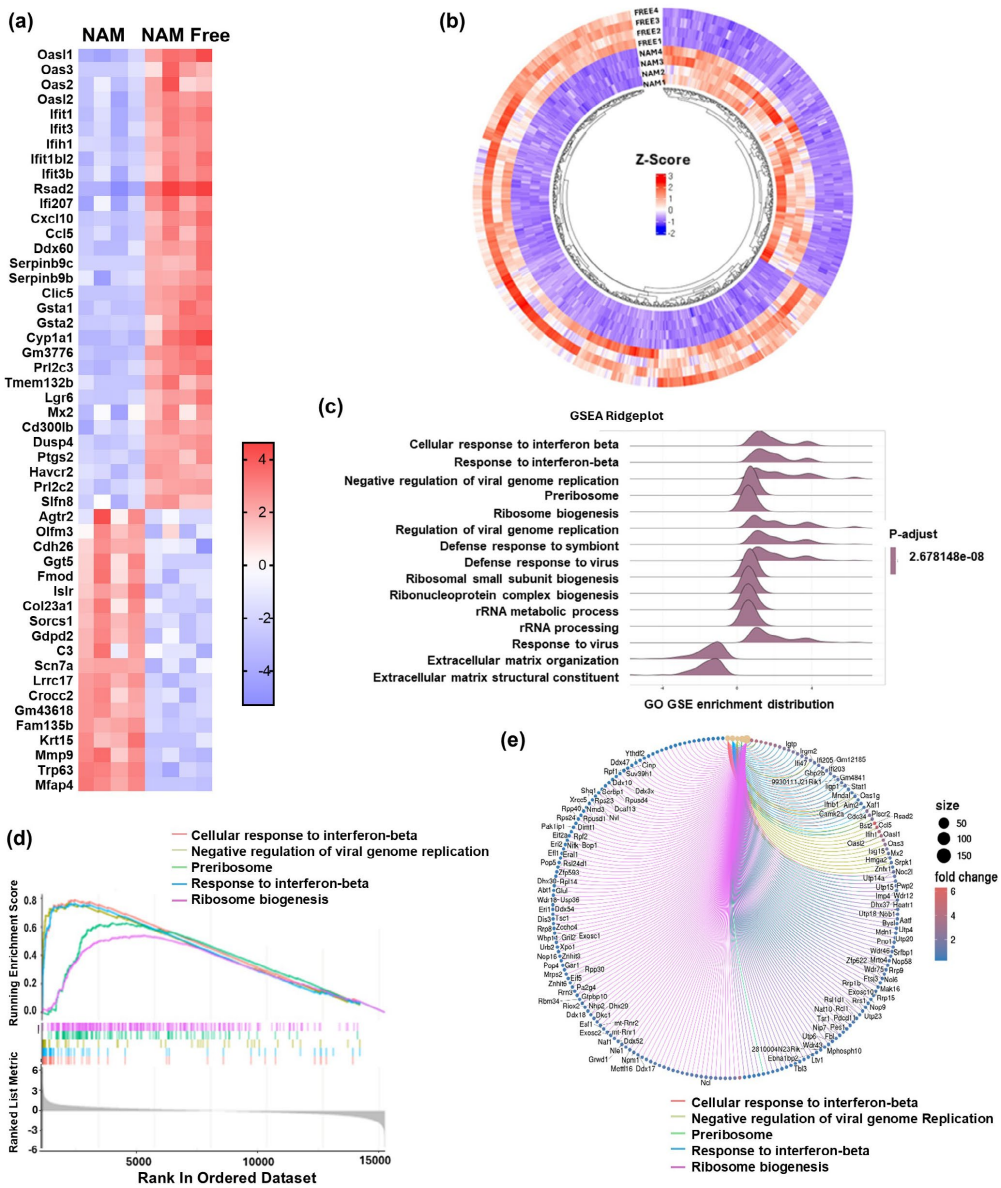
NAM depletion activates an interferon Type I response. NIH3T3 cells were cultured in NAM or NAM Free media for 12 days (*n* = 4). (a) Heat map shows the enrichment of the top 50 genes. (b) Circos heat map shows differentially expressed genes (DEGs). (c) Ridge plot shows the top 15 pathways altered in cells grown in NAM Free media compared to NAM media using GSEA analysis performed on gene ontology (GO) database. (d, e) Top 5 pathways ranked by GSEA analysis from the GO database (d) and cnetplot (e).

To characterize how NAM depletion affects cellular homeostasis and inflammation‐related signaling pathways, we performed RNA sequencing on NIH3T3 cells grown in NAM and NAM Free media. We analyzed four independent experiments, where cells were grown from 11 to 12 days under each condition. Hierarchical clustering of the top 1000 genes revealed that NAM depletion led to significant alterations in gene expression (Figure S4a), with minimal variability among replicates. Interestingly, the top 50 regulated genes included upregulation of several interferon (IFN)‐stimulated genes (*Oasl1/2, Oas2/3, Ifit1, Ifit3, Ifih1*, *Ccl5, Cxcl10, Rsad2, Ddx60*) (Hubel et al. 2019) (Figure 4a). We also found increased expression of genes involved in cell survival against stress, including *Serpinb9* (also known as Proteinase Inhibitor 9) genes, which have been shown to safeguard cells from apoptosis (Huang et al. 2024), and glutathione S‐transferases *Gsta2* and *Gsta*3, which help detoxify cells from oxidative stress (Mazari et al. 2023) (Figure 4a). On the other hand, the most downregulated genes were associated with cell proliferation, apoptosis, and cell cycle regulation, including *Gas7, Lrrc17, Notch3*, and *Trp63*. Additionally, a principal component analysis (PCA) plot of the first and second components clearly distinguished cells cultured in NAM versus NAM Free conditions (Figure S4b), further supporting the significant molecular changes induced by NAM depletion.

With the DESeq2 package, we identified 3765 differentially expressed genes (DEGs) (1649 upregulated genes and 2116 downregulated genes) between cells treated with or without NAM media using a threshold of < 0.05 of false discovery rate (FDR) and fold change of > 1.5. The volcano plot shows that NAM depletion triggered a substantial transcriptomic response (Figure S4c) and the Circos plot shows the hierarchical distribution of DEGs (Figure 4b).

Gene Set Enrichment Analysis (GSEA) was performed to assess the enrichment of predefined gene sets based on Gene Ontology (GO) terms. The Ridge plot displays the top 15 upregulated pathways altered by NAM depletion: cellular response to interferon beta, negative regulation of viral genome replication, response to viruses, preribosome, ribosome biogenesis, and rRNA metabolic processes and processing (Figure 4c). In contrast, pathways such as extracellular matrix organization and extracellular matrix structural constituent were the main downregulated pathways (Figure 4c).

An enrichment score plot was created to illustrate the enrichment score changes based on the absolute value of fold change. Once again, the top five pathways identified by GO GSEA that were altered by NAM depletion were: cellular response to interferon beta, negative regulation of viral genome replication, preribosome, response to interferon beta, and ribosome biogenesis (Figure 4d). To further explore the relationships between genes and their associated gene sets, we generated a cnetplot which visualizes the interactions among the top five altered pathways (Figure 4e).

Finally, tree plots were created for both Gene Ontology (GO) and KEGG pathway enrichment analyses, focusing specifically on DEGs (Figure S4d,e). Overall, our transcriptomic analysis revealed that the Type I interferon response was the top upregulated pathway in NAM‐depleted cells, while extracellular matrix‐related pathways were the top downregulated.

**FIGURE 5:**
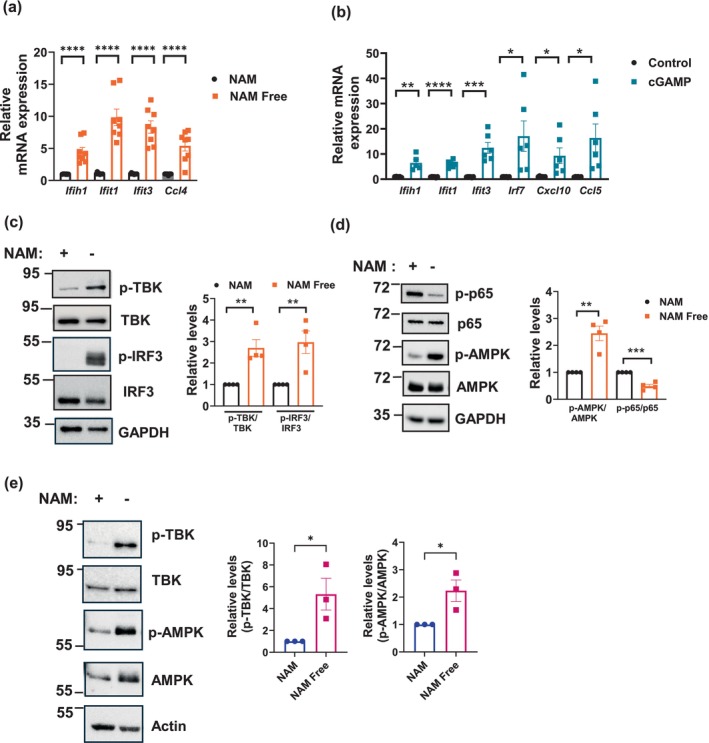
Nicotinamide depletion activates the cGAS‐STING‐interferon pathway. (a) Graph shows the relative expression of interferon‐dependent genes assessed by qPCR in cells cultured for 7 days in NAM and NAM Free media (*n* = 8). (b) qPCR analysis of cells treated with 15 μM cGAMP for 16 h (*n* = 5–6). (c, d) Representative immunoblots of cell lysates of NIH3T3 cells cultured for 9–12 days in NAM and NAM Free media. Graphs show quantification of immunoblots (*n* = 4). (e) Representative immunoblot and quantification of immunoblots from cell lysates of HS5 cells cultured in NAM and NAM Free media for 14 days (*n* = 3). Data are presented as mean ± SEM, with *n* representing the number of different experiments. *p* Values were calculated using unpaired two‐sided *t*‐tests. *P < 0.05, **P < 0.01, ***P < 0.001, ****P < 0.0001.

To validate our RNA seq findings, we conducted qPCR on several interferon‐related genes in NIH3T3 cells cultured in NAM and NAM Free media for 7 days. Cells cultured without NAM showed upregulation of *Ifih1*, *Ifit1*, *Ifit3*, and *Ccl4* (Figure 5a). This aligns with our results in Figure 3a showing increased expression of the interferon‐dependent genes *Cxcl10*, *Ccl5*, and *Irf7* in NAM Free conditions. Notably, this pattern closely resembles the gene expression changes observed after treating NIH3T3 cells with cGAMP, an activator of the cGAS‐STING‐interferon pathway. Cells treated with cGAMP for 16 h displayed increased expression of many interferon‐related genes, whereas no changes were seen in *Il6, Cxcl1*, and *p21* (Figure 5b, Figure S5a). We also confirmed by qPCR the downregulation of several of the most suppressed genes identified by RNA‐seq in NAM depleted cells, such as *Gas7*, *Lrrc17*, *Notch3*, and *Trp63* (Figure S5b). In addition, numerous fibrosis‐related genes, including metalloproteinases Mmp2, Mmp3, Mmp14 and the collagen genes *Col1a* and *Col5a*, were also downregulated under NAM Free conditions, especially at 14 days (Figure S5c), validating our RNA‐seq data.

To further support our findings that NAM/NAD depletion induces an interferon response, we examined the activation of TBK and IRF3, key components of the cGAS‐STING pathway (Chen and Xu 2023). Depletion of NAM for 7–9 days in NIH3T3 cells increased the phosphorylation of both TBK (S172) and IRF3 (S396), compared with nondepleted cells (Figure 5c). We also investigated the impact of NAM depletion on other signaling pathways involved in inflammation and cell survival. NF‐κB is a major regulator of inflammatory responses, and phosphorylation of its p65 subunit appears to be important for activation of this pathway (Christian et al. 2016). In NAM‐depleted cells, there was a decrease in p65 phosphorylation (S536), suggesting inhibition of NF‐κB activation (Figure 5d). This inhibition may account for the reduced expression of *Ccl2*, *Cxcl1*, and *Mmp9* seen in these cells (Figure 3a). These results highlight potentially important differences in two canonical inflammatory responses modulated by NAD metabolism, with the cGAS‐STING pathway activated by NAD collapse, while NF‐kB activation is dependent on maintenance of normal cellular NAD levels.

We also examined the AMPK pathway, a key regulator of metabolism that helps restore energy balance during metabolic stress (Herzig and Shaw 2018). In NAM‐depleted cells, the ratio of p‐AMPK(Thr172)/AMPK increased, indicating activation of this pathway (Figure 5d). Since AMPK is known to inhibit cell growth and proliferation, its activation in NAM‐depleted cells may drive both metabolic and cell growth adaptations.

Activation of the cGAS–STING pathway was also confirmed in IMR90 and HS5 cells, as evidenced by increased p‐TBK/TBK ratio (Figure 5e and Figure S5d,e). Additionally, HS5 cells cultured in NAM‐depleted media showed elevated p‐AMPK/AMPK ratio and increased total protein acetylation, consistent with the AMPK activation and inhibition of deacetylase activity observed in NIH3T3 cells (Figure 5e, Figure S5e,f). Notably, reintroduction of NAM to the cell culture media restored both the p‐TBK/TBK and p‐AMPK/AMPK ratios in HS5 cells to baseline levels (Figure S5e), indicating the reversibility and NAD dependency of these processes.

### 2.4 Nicotinamide Depletion Promotes Mitochondrial Dysfunction and Mitochondrial dsDNA Leakage to the Cytosol

**FIGURE 6:**
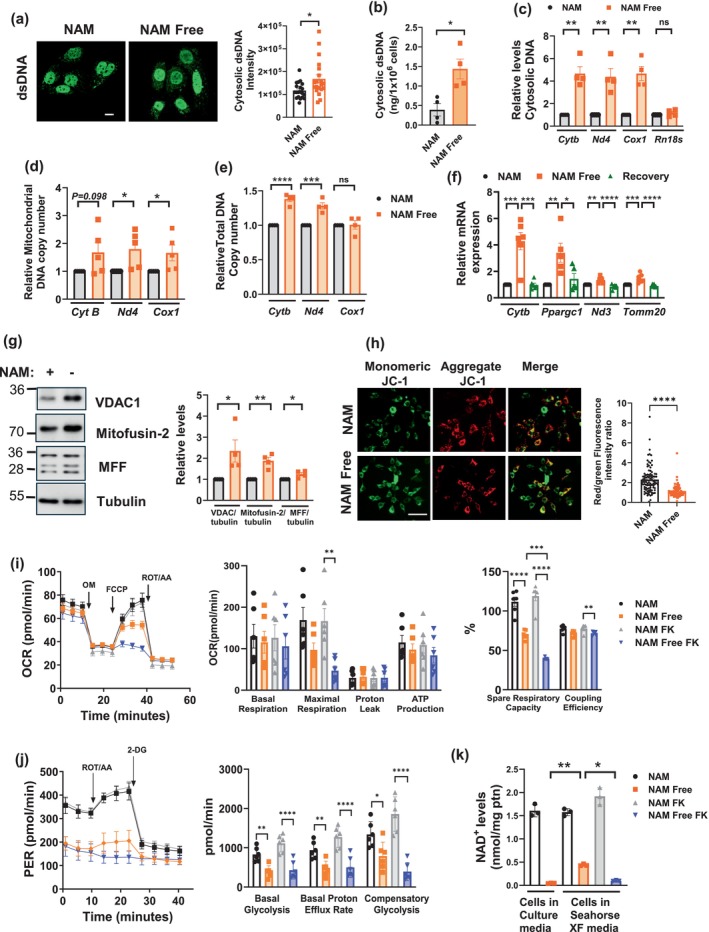
Nicotinamide depletion promotes mitochondrial dysfunction and mitochondrial dsDNA leaking to the cytosol. (a–j) NIH3T3 cells were grown for 8–14 days in NAM or NAM Free media. (a) Immunofluorescence at 8 days in media showing the presence of cytosolic dsDNA and quantification from a representative experiment (scale bar 10 μm). (b, c) After subcellular fractionation at 9 days in media, the cytosolic fraction was used to measure the amount of dsDNA (b) or to assess the presence of cytosolic DNA by qPCR (c) (*n* = 4). (d, e) Mitochondrial DNA copy number measured by qPCR in the mitochondrial fraction (d) and in the total fraction (e). Levels were calculated relative to 18 s in the total DNA fraction and expressed relative to NAM conditions. (f) Cells were grown for 14 days in media with and without NAM, and for 9 days in NAM Free, followed by recovery in NAM media for 5 days. Graphs show relative gene expression assessed by qPCR (*n* = 5–8). (g–k) Cells were cultured for 9 days in NAM and NAM Free media. (g) Immunoblotting shows levels of mitochondrial proteins VDAC1, Mitofusin‐2, and MFF and graph shows quantification (*n* = 4). (h) Fluorescence staining and quantification of a representative experiment with mitochondrial potential marker JC‐1 (scale bar 62.5 μm). The red fluorescence indicates the JC‐1 aggregate, while the green fluorescence indicates the JC‐1 monomer. (i–k) After culture in NAM and NAM Free media, cells were incubated in Seahorse XF media with and without 5 nM FK866 (FK). (i) Mitochondrial oxygen consumption (OCR) was measured using 1.5 μM Oligomycin (OM), 2 μM Carbonyl cyanide‐p‐trifluoromethoxyphenylhydrazone (FCCP) and 0.5 μM Rotenone/Antimycin A (ROT/AA) in the Seahorse analyzer (*n* = 6). (j) Glycolytic rate was measured using ROT/AA and 50 mM 2‐deoxy‐D‐glucose(2‐DG) in the Seahorse analyzer (*n* = 6). (k) NAD^+^ levels were measured from cells collected before (cells in culture media) and after addition of Seahorse XF media for 1 h (*n* = 3). Data are presented as mean ± SEM, with *n* representing the number of biological replicates. *p* Values were calculated using unpaired two‐sided *t*‐tests or one‐way ANOVA. *P < 0.05, **P < 0.01, ***P < 0.001, ****P < 0.0001.

Given that mtDNA released to the cytosol can trigger STING activation in response to microbial products, virus infection, and other stressors (Kim et al. 2023), we investigated whether NAM depletion in fibroblasts leads to mtDNA release into the cytoplasm. First, we assessed the presence of dsDNA in the cytosol of fibroblasts cultured in NAM or NAM Free media. Immunofluorescence with a dsDNA‐specific antibody, in NIH3T3 and IMR90 cells, showed that cells with low NAD levels had higher levels of cytosolic dsDNA compared to controls (Figure 6a, Figure S6a). To confirm these findings, we performed subcellular fractionation and quantified dsDNA levels in the cytosolic fraction of NIH3T3 cells. As shown in Figure 6b, cells cultured in NAM Free media for 7–9 days exhibited a significant increase in cytosolic dsDNA compared with those cultured in NAM media. To identify the source of this dsDNA, we performed qPCR on the cytosolic fraction to detect both mitochondrial‐ and nuclear‐encoded DNA. Under NAM Free conditions, we observed a 3‐ to 5‐fold increase in cytoplasmic mitochondrial‐encoded DNA, including NADH dehydrogenase 1, 3, and 4 (*Nd1, Nd3 and Nd4*), cytochrome *b* (*Cytb*), and cytochrome *c* oxidase I (*Cox1*) (Figure 6c, Figure S6b). In contrast, nuclear‐encoded genes such as ribosomal gene *Rn18s, Tomm20, and Actb* (actin beta) showed little (1.5‐fold) or no change (Figure 6c, Figure S6b), suggesting that the dsDNA leakage is primarily originated from the mitochondria. Importantly, the purity of our subcellular fractionation was confirmed by immunoblotting for cytosolic (GAPDH), mitochondrial (VDAC1), and nuclear (lamin B1 and histone H3) proteins (Figure S6c,d).

In NAM‐depleted cells, we observed that NAD levels were extremely low in both the cytosolic and mitochondrial fractions (Figure S6e,f). Interestingly, a recent study proposed that there is communication between the NAD pools and that the mitochondrial NAD pool can compensate for a deficiency in cytosolic NAD. The authors propose that cellular dysfunction only occurs when the mitochondrial pool is depleted (Høyland et al. 2024). Notably, their study found no major impairments in cellular proliferation and viability, even when mitochondrial NAD was reduced to nearly 50% of control levels. The only observed changes were metabolic adaptations, including changes in mitochondrial function and glycolysis (Høyland et al. 2024). Our data extend these findings, showing that cells can adapt to even more severe depletion of cellular NAD, including mitochondrial NAD, and maintain viability for several weeks. This suggests that cells can undergo significant adaptations to support a dramatic decline in NAD levels.

To further investigate mitochondrial changes in NAD‐depleted cells, we measured the mitochondrial DNA copy number in both isolated mitochondrial fraction and total DNA fraction. Under NAM Free conditions, we found increased copies of the mitochondrial genes *Cytb*, *Nd4*, and *Cox1* in DNA from both isolated mitochondria and total cellular extracts (Figure 6d,e), except for *Cox1* in the total DNA fraction. These data indicate an increased mitochondrial mass in NAM‐depleted cells. The mRNA expression of genes associated with mitochondrial function and biogenesis, such as *Cytb, Ppargc1a, Nd3, and Tomm20*, also showed elevated expression (Figure 6f), along with higher levels of the mitochondrial proteins VDAC1, mitofusin‐2, and Mitochondrial fission factor (MFF) (Figure 6g). Importantly, reintroducing NAM reversed the upregulation of mitochondrial gene expression (Figure 6f). Cell labeling with MitoTracker green confirmed increased live mitochondria in NAM Free cells (Figure S6g). These results together suggest that the absence of NAM leads to an increase in mitochondrial abundance in cells. At the protein level, we also observed decreased levels of the transcription factor TFAM (Figure S6h), which is a pivotal factor for regulating mtDNA replication. Interestingly, decreased levels of TFAM have been shown to affect mtDNA stability, promoting the release of mtDNA into the cytoplasm, triggering the cGAS‐STING pathway (Li et al. 2022).

Since mitochondria play a key role in cellular energy metabolism, and disruptions in their function can lead to inflammation, cell cycle arrest, and apoptosis (Vringer and Tait 2019), we investigated mitochondrial function in these cells. Since mitochondrial membrane potential is closely associated with mitochondrial function, we first measured the mitochondrial membrane potential (MMP) using the JC‐1 probe. The results showed that NIH3T3 cells in NAM Free media had reduced MMP (Figure 6h). Next, we measured mitochondrial respiration and glycolysis using the Seahorse Mito Stress and Glycolytic Rate Assays (Figure 6i,j). In the Mito Stress assay, while basal respiration, ATP production, and proton leak‐coupled respiration remained largely unaffected, maximal respiration and spare respiratory capacity were significantly impaired (Figure 6i). These impairments were further exacerbated by FK866 (Figure 6i), a potent NAMPT inhibitor, which was included during the Seahorse assay to block partial NAD recovery from NAM present in the assay medium (Figure 6k). Blocking this partial NAD recovery (about 25%) uncovered a more pronounced defect in maximal respiration and spare capacity in cells cultured in NAM Free media (Figure 6i,j). These data indicate that although mitochondrial function is impaired in NAM/NAD deficient cells, the basal mitochondrial function and the coupled respiration are mostly preserved, possibly due to the increased mitochondrial number. In contrast, the ability to respond to increased metabolic demand appears to be severely impaired in these cells. Moreover, the data also indicate that the effects of NAD depletion on mitochondrial function can be partially and rapidly reversed by NAD recovery in the presence of NAM‐containing media (Figure 6i–k). Importantly, glycolytic activity was even more sensitive to NAD depletion, with all measured parameters severely reduced (Figure 6j). The modest improvement in glycolysis seen with 25% NAD recovery in NAM‐containing media was limited. Except for compensatory glycolysis, there was no statistically significant difference between NAM Free cells and those treated with FK866 during the glycolytic rate assay (Figure 6j). These findings suggest that mitochondrial oxidative phosphorylation and cytosolic glycolysis may have different sensitivity thresholds to NAD availability.

We also measured reactive oxygen species (ROS) in cells grown in either NAM or NAM Free media. In both conditions, ROS were undetectable. However, when treated with doxorubicin (0.3 μM for 24 h) to promote ROS production, cells with low NAD levels showed significantly lower ROS levels than those with normal NAD (Figure S6i). Since ROS are unstable, we also evaluated lipid peroxidation, a by‐product of ROS. Cells grown in NAM Free media for 9 days exhibited higher levels of malondialdehyde (MDA), a marker of lipid peroxidation, compared to those grown in NAM media (Figure S6j). This increase in lipid peroxidation may be linked to the lower GSH/GSSG ratio observed in NAM‐depleted cells (Figure 2i) and suggests increased oxidative damage in these cells. To evaluate changes in antioxidant defenses in cells cultured in NAM Free media, we measured both the levels and enzymatic activity of two key antioxidant enzymes: catalase and superoxide dismutase (SOD). Although SOD2 protein levels increased under NAM Free conditions, we also observed an increase in SOD2 acetylation (Figure 2g and Figure S6k), which may suggest reduced enzymatic activity (Dikalova et al. 2017). Additionally, neither catalase levels nor activity increased, and total SOD activity remained unchanged (Figure S6k,l). Collectively, these findings suggest that cells in NAM Free media do not regulate the overall activity of these antioxidant enzymes. This lack of response, combined with a reduced GSH/GSSG ratio (Figure 2i), may explain the elevated oxidative damage observed in NAM Free conditions, despite the potential decrease in ROS production during oxidative stress induced by agents such as doxorubicin.

### 2.5 STING and VDAC modulate the inflammatory response induced by NAM depletion

**FIGURE 7:**
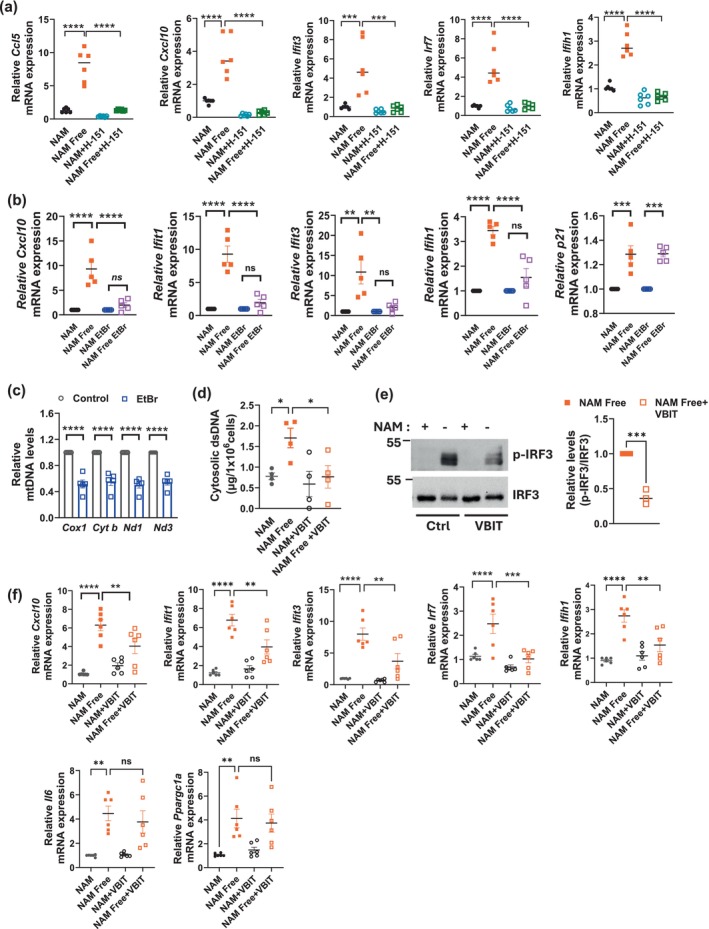
STING, mtDNA, and VDAC regulate the inflammatory response induced by NAM depletion. (a) NIH3T3 cells were cultured in NAM and NAM Free media for 7 days. For the last 24 h, cells were treated with 0.5 μM H‐151 (STING inhibitor). Graphs show qPCR analysis (*n* = 4,6). (b, c) NIH3T3 cells were cultured with ethidium bromide (EtBr) to deplete mtDNA, then cultured in NAM or NAM Free media for an additional 7 days. (b) Gene expression assessed by qPCR (*n* = 5). (c) Mitochondrial DNA copy number measured by qPCR (*n* = 5). (d–f) NIH3T3 cells cultured for 8–12 days in NAM or NAM Free media were treated with or without 5 μM VBIT‐4 (VDAC inhibitor) for the last 48 h. (d) After subcellular fractionation, the cytosolic fraction was used to quantify the amount of dsDNA (*n* = 4). (e) Representative immunoblot and quantification of NAM Free cells cultured with and without VBIT‐4 (*n* = 3). (f) Expression of interferon‐dependent genes assessed by qPCR (*n* = 6). Data are presented as mean ± SEM, with *n* representing the number of different biological replicates. *p* Values were calculated using unpaired two‐sided *t*‐tests or one‐way ANOVA. *P < 0.05, **P < 0.01, ***P < 0.001, ****P < 0.0001.

To confirm whether activation of the cGAS‐STING pathway is necessary for the interferon inflammatory response induced by NAM depletion, we tested the effect of a STING inhibitor, H‐151 (Haag et al. 2018). After culturing NIH3T3 cells for 6 days in NAM Free media, we incubated them for 24 h with 0.5 μM H‐151. This treatment completely reversed the increased expression of interferon‐dependent genes, including *Ccl5, Cxcl10, Ifit3, Irf7*, and *Ifih1* (Figure 7a), keeping *p21* and *Ppargc1a* unaffected (Figure S7a). To further confirm the importance of this pathway, we tested another STING inhibitor, C‐176, in HS5 cells. Like H‐151, C‐176 also blocked the increased expression of interferon genes induced by NAM/NAD depletion, but it did not affect the expression of *Il6* or *Cxcl8* (Figure S7b).

To explore the role of mitochondria in the interferon response induced by NAM depletion, we treated NIH3T3 cells with ethidium bromide to deplete mitochondrial DNA (mtDNA) (Wiley et al. 2016). After confirming a 50% reduction in mtDNA (achieved between 20 and 40 days), cells were cultured in NAM and NAM Free media for 7 days. mtDNA depletion abolished the interferon response induced by NAD depletion, while the upregulation of *p21* expression remained unaffected (Figure 7b,c).

To further investigate the molecular mechanisms involved in the mitochondrial dsDNA leakage induced by NAD depletion, we examined whether the voltage‐dependent anion channel (VDAC) was involved. VDAC is a key protein in the mitochondrial outer membrane that regulates mitochondrial function and the release of mtDNA into the cytosol under certain types of cellular stress (Kim et al. 2023). A recent study found that VBIT‐4, a specific inhibitor of VDAC1, can prevent VDAC1 oligomerization and inhibit mtDNA release into the cytoplasm (Kim et al. 2019). In NIH3T3 cells cultured for 8 days in NAM or NAM Free media, the addition of VBIT‐4 for the last 24 h significantly reduced dsDNA leakage, phosphorylation of IRF3, and the expression of several interferon‐dependent genes under NAM Free conditions (Figure 7d–f). In contrast, VBIT‐4 had no effect on the expression of *Il6* and *Ppargc1a* induced by NAM depletion (Figure 7f), suggesting these genes are regulated independently of mtDNA leakage. These results indicate that mtDNA leakage through VDAC oligomerization is necessary for the activation of the cGAS‐STING pathway and triggering the interferon response induced by NAM/NAD depletion.

## 3 Discussion

NAD is one of the most ancient bioenergetic molecules, involved in a wide range of biochemical pathways from energy metabolism to DNA repair and cellular signaling (Amjad et al. 2021; Dherbassy et al. 2024). NAD metabolism is highly dynamic, with cellular NAD levels typically ranging from high micromolar to millimolar concentration (Migaud et al. 2024). NAD depletion has evolved as one of the primordial mechanisms in response to infections (Garb et al. 2022; Wang et al. 2023). For instance, bacteria can promote NAD decline to limit phage infection and proliferation (Garb et al. 2022; Osterman et al. 2024; Wang et al. 2023). Moreover, NAD decline is an important feature of the interaction between infectious agents and the immune system of higher organisms (Hogan et al. 2019; Tan and Doig 2021; Tang et al. 2024).

In addition to its role in immune responses, NAD decline is implicated in multiple disease states and during injury and aging (Chini et al. 2023; Doke et al. 2023; Lautrup et al. 2024; Strømland et al. 2021). Since humans depend on dietary sources of precursors, including forms of vitamin B3, to synthesize NAD, even transient deficiencies in vitamin B3 can lead to low NAD levels, significantly impacting disease states, healthspan, and longevity (Chini et al. 2021; Feuz et al. 2023). However, the cellular consequences of chronic NAD depletion are not well defined, and significant questions remain unanswered regarding the adaptations that occur in response to decreased NAD levels. For example, it is still unknown how much NAD is required for a cell to sustain its basic metabolic functions, maintain cellular integrity, and preserve its proliferative capacities. Furthermore, it is unclear which specific pathways are regulated by low NAD states that could influence cellular, tissue, and organismal function. These uncertainties highlight the importance of understanding the mechanisms by which cells respond and adapt to NAD deficiency.

Surprisingly, we found that fibroblasts can tolerate extremely low NAD levels for extended periods of time, even when mitochondrial NAD levels were severely depleted. In our study, chronic depletion of NAM led to persistently low levels of NAD, NADH, and several other NAD metabolites. In fact, after 2 days in NAM Free media, NAD collapsed to extremely low levels and continued to stay low for the duration of the experiments (usually between 7 and 14 days). Despite this, there was only a small decrease in the cell growth rate and no significant effects on the cell cycle, apoptosis, or cellular senescence. Regarding mitochondrial function, we observed a decrease in mitochondrial membrane potential, along with significant reductions in spare respiratory capacity and maximal respiration. This indicates that while the basal metabolic activity was maintained in NAM Free conditions, the cells lacked the ability to meet increased metabolic demands. Glycolytic function was even more affected, as cells cultured in NAM Free showed a marked decrease in all glycolytic rate parameters measured. These results indicate that fibroblasts can maintain basic cellular metabolic functions with NAD levels at least 20 times lower than normal. Moreover, they suggest that mitochondrial oxidative phosphorylation and cytosolic glycolytic metabolism may have different sensitivity thresholds to NAD depletion.

An intriguing aspect of our study is the identification of the proinflammatory phenotype induced by NAD depletion. This response appears to be mediated by an active mechanism involving mitochondrial VDAC‐mediated mitochondrial DNA leakage to the cytoplasm, which activates the cGAS‐STING pathway. Importantly, NAD decline triggered increased expression of gene sets associated with viral infections, which is particularly interesting given that cells typically respond to viral infections by activating NADases, enzymes that consume NAD, in order to limit viral replication (Shang et al. 2021; Tan and Doig 2021). Moreover, there is also a link between viral responses, cGAS‐STING, and NAD consumption (Morehouse et al. 2020). Many bacterial STING proteins are fused to a toll‐interleukin‐1 receptor (TIR) domain, which has NADase activity (Morehouse et al. 2020). Bacterial STING is activated by 3′3′‐c‐di‐GMP, a common signaling molecule, which induces STING oligomerization and NADase activity. By degrading NAD, the STING‐TIR protein induces energetic catastrophe to kill the viral‐infected cells (Morehouse et al. 2020). While mammalian STING does not appear to have a TIR domain and NADase activity (Chen and Xu 2023), our data suggest that NAD decline itself can activate the cGAS‐STING in mammalian cells, albeit in the opposite direction. In the context of viral infections, NADases such as CD38 or PARPs may promote NAD decline, facilitating leakage of mtDNA and amplifying the cGAS‐STING response.

Analysis of our RNA seq data from NAM‐depleted cells revealed increased expression of several genes associated with RNA sensing, including *Ddx58, Ddx60, Ifih1*, and *Oas1*. The release of mitochondrial double‐stranded RNA (mt‐dsRNA) into the cytosol is known to also activate innate immune signaling pathways, such as the interferon response. For instance, in senescent cells, mtRNA leakage activates cytosolic RNA sensors like RIG‐I (encoded by *Ddx58*) and MDA5 (encoded by *Ifih1*), triggering the senescence‐associated secretory phenotype (SASP) (López‐Polo et al. 2024). Notably, preventing the release of mitochondrial DNA or RNA into the cytosol has been shown to suppress the SASP (López‐Polo et al. 2024; Victorelli et al. 2023). These findings suggest that NAD depletion might also promote mtRNA leakage, a possibility that needs to be explored in future studies.

Interestingly, RNA‐seq analysis of adipocytes isolated from white adipose tissue (WAT) of a Nampt knockout mouse model showed increased expression of several interferon‐related genes—many of which were also upregulated in our RNA‐seq analysis of NIH3T3 cells under NAM Free conditions (Basse et al. 2023). These findings suggest that NAD^+^ depletion in vivo activates interferon‐related signaling pathways, at least in WAT.

In addition to triggering an inflammatory response, NAM depletion in cells also regulated the activation of several proteins involved in cell signaling and metabolic regulation, including sirtuins, PARPs, AMPK, and NF‐κB. The inhibition of sirtuins and PARP activity is expected, as both are NAD‐dependent enzymes, and their activities are directly influenced by NAD availability. AMPK can be activated through multiple mechanisms, including shifts in intracellular AMP, ADP, and ATP levels, as well as through noncanonical pathways such as mitochondrial dysfunction and reactive oxygen species (ROS) (Hinchy et al. 2018; Zhao et al. 2016). The increased AMPK activation observed in our study aligns with previous findings showing that AMPK responds to mitochondrial stress and plays a critical role in metabolic regulation and cell survival under energy‐limiting conditions (Herzig and Shaw 2018; Zhao et al. 2016). Interestingly, a recent study using a cellular model of mitochondrial disease, characterized by progressive mitochondrial DNA (mtDNA) depletion and deteriorating mitochondrial metabolism, shows that mitochondria‐associated AMPK becomes activated early during the advancing mitochondrial dysfunction, before any quantifiable decrease in the ATP/(AMP + ADP) ratio or respiratory chain (Carvalho et al. 2025). Whether mtDNA leakage, AMPK activation, and the interferon response are mechanistically linked in our NAM depletion model remains to be determined. As for NF‐κB, its activation has been proposed to be regulated by the cGAS‐STING pathway (Chen et al. 2016). However, in NAM‐depleted cells, we observed reduced phosphorylation of the p65 subunit of NF‐κB. This suggests that unlike cGAS‐STING, NF‐κB activation may depend on sufficient NAM/NAD levels. Future studies will be crucial to elucidate how these signaling pathways are regulated and how they interact to maintain cell survival under conditions of NAM/NAD depletion.

In cells cultured in NAM‐depleted media, we observed signs of oxidative stress, evidenced by a reduced GSH/GSSG ratio and increased lipid peroxidation. Despite this, there was no indication of an enhanced antioxidant response. SOD2 acetylation increased, indicating reduced activity. Also, there were no increases in catalase expression, catalase activity, or total SOD activity. These results suggest that under NAM‐depleted conditions, cells experience oxidative stress without mounting a sufficient antioxidant defense, potentially leading to the accumulation of oxidative damage.

Importantly, a recent study induced chronic NAD deficiency by overexpressing PARP in specific cellular compartments (Høyland et al. 2024). Compared with control cells, the total cellular NAD^+^ content in PARP1‐expressing cells was reduced to varying degrees (Høyland et al. 2024). The most significant decrease in NAD^+^ levels (about 50%) occurred when PARP1 was expressed in mitochondria and peroxisomes. In line with our study, NAD depletion was surprisingly well tolerated by cells, with no significant impairment in cell viability or growth (Høyland et al. 2024). However, only mitochondrial NAD^+^ depletion led to a decrease in mitochondrial respiration, increased glycolysis, and the accumulation of medium chain fatty acids, without changes in ATP levels (Høyland et al. 2024). The authors proposed that the mitochondrial NMNAT3 enzyme, along with the SLC25A51 transporter, helps maintain cytosolic NAD^+^ by producing and exporting mitochondrial NAD^+^ to the cytoplasm (Høyland et al. 2024). Our findings support this notion and demonstrate that even when NAD^+^ levels are nearly undetectable in both cytosolic and mitochondrial fractions, cells still maintain significant viability. The authors of the aforementioned study suggest that their results are consistent with a well‐tolerated age‐related decline in NAD^+^, provided the mitochondrial NAD pool remains unaffected (Høyland et al. 2024). Therefore, it is possible that the DNA leakage and the viral‐like response observed in our study were mediated by mitochondrial NAD depletion. However, it is also important to highlight that even if cells tolerate NAD depletion well, the activation of a viral‐like response could significantly contribute to cellular and tissue dysfunction during low NAD states. Therefore, the mechanism by which NAD decline promotes dysfunction may involve adaptive responses and signaling pathways modulated by low NAD, rather than solely mediated by an energy deficit at the cellular level.

Our findings offer new insight into how cells sense and respond to NAD depletion and may have broad implications for various diseases, tissue injury, and conditions such as pellagra and aging. Although the extent of NAD depletion in our model exceeds levels typically reported in whole tissues during disease or aging, similar dramatic reductions have been observed in specific in vivo contexts—such as WAT, cochlea, and ovaries of aged mice, as well as in mouse models of acute kidney injury (Basse et al. 2021; Doke et al. 2023; Okur et al. 2023; Yang et al. 2024). Moreover, the degree of NAD decline within individual cell types under these in vivo conditions remains poorly understood. It is possible that similar levels of depletion occur transiently or are more pronounced in certain cell populations during disease progression or aging.

In conclusion, our data indicate that while cells subjected to significant NAD^+^ depletion, including depletion in the mitochondrial pool, can still maintain basic functions and remain viable, the leakage of mitochondrial DNA to the cytoplasm may have a crucial role in the development of an inflammatory response. Therefore, here we have identified that NAD decline triggers a viral‐like response driven by mitochondrial dysfunction‐induced cytosolic DNA leakage through the VDAC mitochondrial pore. This novel mechanism induced by NAD decline may contribute to chronic inflammation in pathological conditions and NAD‐deficient states.

## 4 Materials and Methods

### 4.1 Reagents

The following reagents were used: trichloroacetic acid (TCA) (Sigma‐Aldrich Inc., 271704), sodium hydroxide (EMD Millipore, 567530), 1,1,2‐trichloro‐1,2,2‐trifluoroethane (Spectrum, TR113), trioctylamine (Sigma‐Aldrich Inc., T81000), Tris base (EMD Millipore, 648310), 1‐alcohol dehydrogenase (Sigma‐Aldrich Inc., A3263‐75KU), diaphorase (Sigma‐Aldrich Inc., D5540), riboflavin (Sigma‐Aldrich Inc., F8399), resazurin (Sigma‐Aldrich Inc., 199303), Trichostatin A (TSA) (Sigma‐Aldrich Inc., T1952), gallotannin (Enzo, ALX‐270‐418), NAM (Sigma‐Aldrich Inc., 9892‐0), NMN (nicotinamide mononucleotide, Sigma‐Adrich 1094‐61‐7), NR (nicotinamide riboside, Laurus Laboratory). When not specified, reagents and chemicals were purchased from Sigma‐Aldrich.

### 4.2 Cell Culture

The fibroblast cell lines NIH3T3 (mouse, isolated from NIH/Swiss embryo) and IMR90 (normal human fibroblasts derived from lung tissue), and HS5 cells (human, derived from bone marrow stromal cells) were from ATCC. NIH3T3 and IMR90 cells were cultured in Dulbecco's modified Eagle medium (DMEM, Invitrogen, 11995‐065), while HS5 cells were cultured in RPMI (Invitrogen, 11875093). Both media were supplemented with 10% fetal bovine serum (FBS, Gibco, A56708‐01) and 1% penicillin–streptomycin (Life Technologies, 15140‐122). To promote NAM depletion, NIH3T3, HS5, and IMR90 cells were cultured in DMEM media without nicotinamide (NAM Free). This custom‐ordered media was based on DMEM 11995‐065 (Gibco) and specifically prepared without nicotinamide. It was supplemented with 10% dialyzed FBS (Gibco, 26400‐044) and 1% penicillin–streptomycin (Life Technologies, 151‐40‐122). For cells cultured in the presence of NAM, 30 μM was added to the NAM Free media. Cells were cultured for specific durations (between 7 and14 days) in NAM and NAM Free media, which are specified in the corresponding figure legend. For recovery experiments, cells grown in NAM Free media for several days were incubated again in media containing 30 μM NAM, NMN, or NR for 3–5 days.

### 4.3 Drug Treatments

Cells in NAM or NAM Free media were treated with the following drugs: 5 μM VBIT‐4 (Selleckchem, S3544), 1.5 μM H151 (InvivoGen, inh‐h151), 3 μM C‐176 (Selleckchem S6575), 15 μM 2′3′‐cGAMP (APExBIO, 1441190‐66‐4), 5 nM FK866 (Sigma‐Aldrich Inc., F8557), and 250 μM phthalic acid (Sigma‐Aldrich Inc., P2944). The duration of each treatment is specified in the corresponding figure legend.

### 4.4 NAD ^+^ Levels by Cycling Assay

NAD^+^ levels were measured as previously described using a cycling assay (Camacho‐Pereira et al. 2016; Kanamori et al. 2018). Cells were homogenized in 10% trichloroacetic acid (TCA) using a sonicator (Fisherbrand model CC‐18). Samples were centrifuged at 12,000 r.p.m. for 2 min at 4°C. The supernatants were collected, and the pellets were resuspended in 0.2 N NaOH for protein determination. TCA was extracted with organic solvents (three volumes of 1,1,2‐trichloro‐1,2,2‐trifluroethane: one volume of trioctylamine) in a ratio of two volumes of organic solvent to one volume of sample. After phase separation, the top aqueous layer containing NAD^+^ was recovered, and the pH was corrected by the addition of 1 M Tris (pH 8.0). For the cycling assay, samples were diluted in 100 mM sodium phosphate buffer (pH 8) in a volume of 100 μL per well and added to white 96‐well plates. Next, 100 μL of reaction mix (0.76% ethanol, 4 μM flavin mononucleotide, 27.2 U mL^−1^ alcohol dehydrogenase (ADH), 1.8 U mL^−1^ diaphorase and 8 μM resazurin) was added to each well. Then, 96‐well plates were read in a fluorescence plate reader (Molecular Devices, SpectraMax Gemini XPS) at an excitation wavelength of 544 nm and an emission wavelength of 590 nm.

### 4.5 NAM, NR, ADPR, and NMN Measurements by HPLC‐MS

NAD precursors and metabolites were measured as described (Tarragó et al. 2018). The HPLC was at a flow rate of 0.25 mL min^−1^ with 99% buffer A from 0 to 3 min, a linear gradient to 99% buffer A/1% buffer B (100% methanol) from 3 to 20 min, 80% buffer A/20% buffer B from 20 to 21 min, a linear gradient to 30% buffer A/70% buffer B from 21 to 28 min at 0.35 mL min^−1^, 99% buffer A/1% buffer B from 28 to 31 min and a linear gradient to 99% buffer A from 31 to 37 min at 0.25 mL min^−1^. Concentrations were quantified based on the peak area compared to a standard curve and normalized to protein content in the sample.

### 4.6 β‐Galactosidase Activity Determination by Flow Cytometry

β‐galactosidase activity was assessed using the CellEvent Senescence Green Detection Kit (Invitrogen, C10850). A total of 1 × 10^6^ cells were washed twice with PBS (with centrifugation at 500 *g* for 5 min at 4°C) and then fixed for 10 min at room temperature, protected from light. Cells were washed twice with 500 μL of 1% BSA in PBS to remove the Fixation Solution. Then cells were resuspended in 200 μL of prewarmed Working Solution and incubated for 2 h at 37°C without CO_2_, protected from light. After incubation, cells were washed three times with 500 μL of PBS and resuspended in 500 μL of Cell Staining Buffer (Biolegend, 420201). Analysis was performed using an Attune NxT Flow cytometer and analyzed using FlowJo software.

### 4.7 β‐Galactosidase Staining

Cells were stained using the Senescence detection kit following the manufacturer's instructions (Abcam, 65351).

### 4.8 Apoptosis Assay

Apoptosis was determined using the FITC Annexin V Apoptosis Detection Kit with PI (Biolegend, 640914). Cells were trypsinized, washed twice with cold Cell Staining Buffer, and then resuspended at 1.0 × 10^6^ cells in 100 μL of Annexin V Binding Buffer with 5 μL of FITC Annexin V. Cells were incubated for 15 min at room temperature (25°C) protected from light. Subsequently, 400 μL of Annexin V Binding Buffer and 10 μL of Propidium Iodide Solution were added. Analysis was performed using an Attune NxT Flow cytometer and analyzed using FlowJo software.

### 4.9 Cell Cycle Analysis

Cell cycle was analyzed via flow cytometry. A total of 1.0 × 10^6^ cells were washed twice with cold PBS and fixed in 1 mL of cold 70% ethanol on ice for 30 min. Cells were washed twice with cold PBS, then treated with 10 μL of RNase A (Thermo Scientific, EN0531) and 10 μL of Propidium Iodide Solution in 80 μL of Cell Staining Buffer, followed by incubation at room temperature for 10 min, protected from light. Prior to analysis, 400 μL of Cell Staining Buffer was added. Analysis was performed using an Attune NxT Flow cytometer and analyzed using FlowJo software.

### 4.10 Growth Rate Calculation

Cells were cultured in NAM and NAM Free media for up to 10 days. Cells in NAM and NAM Free were counted on Days 4, 7, and 10 using Trypan Blue reagent (Gibco, 1525001). On Days 4 and 7, cells in NAM and NAM Free were replated at the same density. Growth rate on Days 4, 7, and 10 was calculated using the formula μ = ln(*n*_1_/*n*_0_) × 24 h/Δ*t*, where μ = growth rate [1/day]; Δ*t* = hours of growth [h], *N*_0_ = number of cells seeded, *N*_t_ = number of cells harvested, and *t*_d_ = doubling time [h].

### 4.11 EdU Incorporation Assay

Cells were incubated with 20 μM 5‐ethynyl‐2′‐deoxyuridine (EdU) overnight in a humidified incubator (37°C, 5% CO_2_). Following incubation, cells were processed according to the Click IT‐Edu Alexa Fluor 488 protocol (Invitrogen, c10350). Images were captured using an EVOS FL Auto 2 microscope at 20× magnification.

### 4.12 Immunoblotting

Immunoblotting was performed as previously described (Tarragó et al. 2018). Cells were lysed in NETN buffer (20 mM Tris–HCl (pH 8.0), 100 mM NaCl, 1 mM EDTA and 0.5% Nonidet P‐40) supplemented with 50 mM β‐glycerophosphate, 5 mM NaF, and a protease inhibitor cocktail (Roche, 11836170001). To detect protein acetylation, the lysis buffer was supplemented with the deacetylase inhibitors nicotinamide (5 mM) and TSA (5 μM). For parylation detection, 100 μM gallotannin (PARG inhibitor) was added to the buffer. After 20 min incubation on ice, samples were centrifuged at 12,000 r.p.m. for 10 min at 4°C. Protein concentrations were determined in supernatants using the Bio‐Rad protein assay (5000006). Lysates were separated by SDS–PAGE and transferred to polyvinylidene difluoride (PVDF) membranes (Immobilon‐P; Millipore). After incubation with specific primary and secondary antibodies, enhanced chemiluminescence detection was performed using SuperSignal West Pico or Femto Chemiluminescence Substrate (Thermo Scientific). Images were captured using Amersham Imager 600 and processed using ImageJ. A list of antibodies used for immunoblotting, along with their respective dilutions, is provided in Table S1.

### 4.13 Metabolomics Analysis

Samples were processed and analyzed by the Metabolomic Core from Mayo Clinic Rochester, following the methodology for qualitative large‐scale profiling. Briefly, cell pellets (3.5 × 10^6^ cells) were lysed in PBS, deproteinized with six volumes of cold acetonitrile: methanol (1:1 ratio), kept on ice with intermittent vortexing for 30 min at 4°C, then centrifuged at 18,000 *g*. ^13^C_6_‐phenylalanine (3 μL at 250 ng μL^−1^) was added as an internal standard to each sample prior to deproteinization. The supernatants were divided into 2 aliquots and dried for analysis on a Quadrupole Time‐of‐Flight Mass Spectrometer (Agilent Technologies 6550 Q‐TOF) coupled with an Ultra High Pressure Liquid Chromatograph (1290 Infinity UHPLC Agilent Technologies). Profiling data were acquired under both positive and negative electrospray ionization conditions over a mass range of 100–1200 *m*/*z* at a resolution of 10,000–35,000 (separate runs) in scan mode. Metabolite separation was achieved using two columns of different polarities: a hydrophilic interaction column (HILIC, ethylene‐bridged hybrid 2.1 × 150 mm, 1.7 μm; Waters) and a reversed‐phase C18 column (high‐strength silica 2.1 × 150 mm, 1.8 μm; Waters). The HILIC column run time was 18 min, and the C18 column run time was 27 min, with a flow rate of 400 μL min^−1^. Each sample was run four times to maximize metabolite coverage. Samples were injected in duplicate, or triplicate as needed, and a pooled quality control (QC) sample, composed of all study samples, was injected multiple times throughout the run. A separate plasma QC sample was also analyzed alongside the pooled QC sample to account for analytical and instrumental variability. Dried samples were stored at −20°C until reconstitution. The samples were reconstituted in running buffer and analyzed within 48 h. Auto‐MS/MS data were also collected using a pooled QC sample to assist in identifying unknown compounds based on their fragmentation patterns. Data alignment, filtering, univariate, multivariate statistical, and differential analysis were performed using Mass Profiler Professional (Agilent Inc., USA). Metabolites detected in at least ≥ 80% of one of two groups were selected for differential expression analyses. Metabolite peak intensities and differential regulation of metabolites between groups were determined. Each sample was normalized to the internal standard and log 2 transformed. An unpaired *t*‐test with multiple testing correction *p* < 0.05 was used to find the differentially expressed metabolites between two groups. Default settings were used, with the exception of the signal‐to‐noise ratio threshold (3), mass limit (0.0025 units), and time limit (9 s). Putative identification of each metabolite was done based on accurate mass (*m*/*z*) against the METLIN database using a detection window of ≤ 5 ppm. The putatively identified metabolites were annotated as Chemical Abstracts Service (CAS), Kyoto Encyclopedia of Genes and Genomes (KEGG), Human Metabolome Project (HMP) database, and LIPID MAPS identifiers (LM_ID).

### 4.14 mRNA Quantification

RNA was isolated using a RNeasy kit (Qiagen, 74136). cDNA was synthesized using the cDNA Reverse Transcription kit (Applied Biosystem, 4368813). Real‐time qPCR was performed using commercially available TaqMan gene expression probes (Applied Biosystems), according to the manufacturer's instructions on a Bio‐Rad CFX384 thermal cycler. The relative mRNA abundance of target genes was calculated by the 2(−ΔΔ*C*_q_) method. Expression changes were calculated relative to cells in NAM media. TaqMan probes used in this study are described in Table S2.

### 4.15 Immunofluorescence

Cells grown in coverslips were fixed with 4% paraformaldehyde (PFA) and permeabilized in 0.2% Triton X‐100 in PBS. After blocking in 2% BSA, coverslips were incubated with dsDNA antibody (Abcam 7156, 1/300) overnight, followed by incubation with antimouse Alexa fluor 488 (Thermo Fisher Scientific, A11001, 1:2000 dilution) and Hoechst 33342 costaining (Invitrogen, 62249). Confocal images were taken using a Zeiss LSM 880 confocal microscope at 60× magnification. Images were processed using ImageJ software.

### 4.16 Cytokines Detection

Cytokines and chemokines were measured using the Bio‐Plex Pro Mouse Chemokine Panel (Bio‐Rad, 12022794) on a Luminex 200 system. First, 50 μL of 1× diluted Bio‐Plex coupled beads were added to each well of a 96‐well plate, followed by two washes with wash buffer. Next, 50 μL of samples and standards were added to the plate, which was then incubated on a shaker at 850 rpm at room temperature for 30 min. After three washes, 25 μL of 1× detection antibody was added to each well, and the plate was incubated again at room temperature for 30 min with shaking. Following another three washes, 50 μL of 1× Streptavidin‐Phycoerythrin (SA‐PE) was added to each well, and the plate was incubated for 10 min at room temperature with shaking. The beads were washed three more times and resuspended in 125 μL of assay buffer. The plate was then analyzed on the Luminex 200 system using MasterPlex software. Data were processed and analyzed using MasterPlex and GraphPad Prism.

### 4.17 RNA‐seq Data Generation, Processing, and Analysis

Four different experiments were performed where NIH3T3 cells were grown for 11–12 days in media with or without NAM. RNA sequencing was performed by MedGenome Inc. (Foster City, CA) using Illumina TruSeq Stranded mRNA Library Prep Kit and running the pooled libraries on NovaSeq sequencer with average read length of 100. Greater than 20 million reads of ~100 bp were generated per sample. The paired‐end reads were aligned to the reference mouse genome downloaded from Ensembl database (genome‐build GRCm38.p6; genome‐version GRCm38; genome‐build‐accession NCBI: GCA_000001635.8) using Spliced Transcripts Alignment to a Reference (STAR, 2.7.3). The aligned reads were used for estimating expression of the genes. The raw read counts were estimated using HTSeq (v0.11.2). Read count data were normalized using DESeq2 (Rajani et al. 2017). Additionally, RNA‐Seq detailed quality control was performed using RNA‐SeQC (v1.1.8), RSeQC (v3.0.1) and MultiQC (v1.7). org.Mm.eg.db package was used for mouse genome annotation. To visualize differences between the NAM and NAM Free groups, hierarchal clustering and principal component analyses were performed using iDEP software (Ge et al. 2018).

Detection of differentially expressed (DE) genes was done using DESeq2. A *p* value cutoff of 0.05 was applied to identify statistically significant changes in gene expression. A fold change greater than 1.5 (in absolute terms) was used to determine biologically significant changes in gene expression. GSEA, pathway enrichment and plotting GO, KEGG, tree plots analysis was done using clusterProfiler 4.0 (Wu et al. 2021). A Circos plot was constructed to visualize the hierarchical distribution of DE genes (Gu 2022; Gu et al. 2016). Each DE gene normalized expression value was converted into *Z*‐scaled values to represent the magnitude and direction of expression changes relative to the mean expression. The *Z*‐scores are visualized as colored segments, providing a comprehensive view of genomic clusters with significant expression changes and allowing for the assessment of grouped patterns of DE genes.

Gene Set Enrichment Analysis (GSEA) was employed to evaluate the enrichment of predefined gene sets (GO terms) in the ranked list of genes. A cnetplot was generated to depict the network of gene sets obtained from the GSEA results. A Ridge plot was created to display the distribution of enrichment scores for all gene sets across the GSEA results. The *x*‐axis represents the enrichment scores, while the *y*‐axis lists the gene sets. Tree plots were produced for both Gene Ontology (GO) and KEGG pathway enrichment analyses, specifically focusing on DE genes. The GO tree plot illustrates the hierarchical structure of GO terms, highlighting how different terms are related and how DE genes contribute to each term. The KEGG tree plot similarly displays the hierarchical organization of KEGG pathways.

### 4.18 Seahorse XF Mito Stress Test and Glycolytic Rate Assay

NIH3T3 cells were seeded at a density of 2 × 10^4^ cells per well in a 96‐well Seahorse XF assay plate 1 day prior to the assay. The Seahorse XF sensor cartridge was hydrated overnight with Seahorse XF Calibrant (Agilent, 100840‐000) at 37°C in a non‐CO_2_ incubator for proper equilibration. On the day of the assay, the culture medium was replaced with 180 μL of prewarmed Seahorse XF DMEM (Agilent, 103575‐100) supplemented with 2 mM glutamine, 25 mM glucose, and 5 nM FK866 (added in some experimental conditions). Assay reagents were prepared using the Seahorse XF Cell Mito Stress Test Kit (Agilent, 103015‐100) and Seahorse XF Glycolytic Rate Assay Kit (Agilent, 103344‐100). The following final concentrations of inhibitors were used: 1.5 μM Oligomycin, 2.0 μM FCCP, and 0.5 μM Rotenone/Antimycin A for the Mito Stress Test; 0.5 μM Rotenone/Antimycin A and 50 mM 2‐Deoxy‐D‐glucose (2‐DG) for the Glycolytic Rate Assay. Following calibration of the sensor cartridge, the cell plate was loaded into the Seahorse XFe96 Extracellular Flux Analyzer. The tests were performed using the Agilent Wave software with the following measurement cycle: three measurements per step, consisting of 2 min mixing followed by 2 min of OCR/ECAR measurement. Data acquisition and analysis were conducted using Agilent Wave and GraphPad Prism software.

### 4.19 Mitotracker Green Staining

Cells cultured in NAM or NAM Free media were incubated for 40 min at 37°C with 250 nM MitoTracker Green (Invitrogen, M7514) in NAM or NAM Free media without FBS. After a wash with PBS, fresh media was added, and images were taken using the GFP filter on an EVOS FL Auto 2 microscope at 40× magnification. Fluorescence intensity was quantified using ImageJ software. The integrated density of fluorescence was measured from 3 to 5 images per condition, depending on cell density. The number of cells analyzed was kept consistent between the NAM and NAM Free conditions.

### 4.20 JC1 Staining

Cells were incubated for 20 min at 37°C with 2 μM JC‐1 dye (Molecular Probes, M34152) in MEB buffer (150 mM Mannitol, 10 mM HEPES, 5 mM Succinate, 50 mM KCl, 0.02 mM EGTA, 0.02 mM EDTA, 0.1% BSA). Following incubation, cells were washed with PBS and fresh MEB buffer was added. JC‐1 accumulates in mitochondria in a membrane potential–dependent manner, shifting its fluorescence emission from green (529 nm, monomeric JC‐1) to red (590 nm, aggregate JC‐1). Images were captured on an EVOS FL Auto 2 microscope at 40× magnification. Fluorescence intensity was quantified using ImageJ software. The integrated density of fluorescence was measured from 3 to 5 images per condition in each experiment, depending on cell density. The number of cells analyzed was kept consistent between the NAM and NAM Free conditions.

### 4.21 Cellular Fractionation

Cells were fractionated into cytosolic, mitochondrial, and nuclear fractions according to the manufacturer's protocol (Thermo Fisher, 89874) with minor modifications. 1–3 × 10^6^ cells were used, and the buffer volumes were adjusted accordingly. The cytosolic fraction was used to quantify cytosolic DNA using the Quant‐iT PicoGreen dsDNA Reagent and Kits (Invitrogen, P7589). Briefly, after isolating the cytosolic fraction, DNA was purified using the Monarch PCR & DNA Cleanup Kit (NEB #T1130). The samples, along with a lambda DNA standard, were incubated with PicoGreen dsDNA Reagent, and fluorescence was measured with excitation at ~480 nm and emission at ~520 nm. The cytosolic and mitochondrial fractions were used to measure NAD^+^ levels, perform immunoblotting, and isolate DNA for qPCR analysis. Total cell lysates were also collected to isolate RNA and DNA for quantification of mRNA transcripts and DNA copy number, respectively, using qPCR. The purity of the subcellular fractions was verified by immunoblotting using antibodies against VDAC1 (mitochondria), GAPDH (cytosol), and lamin B1 and histone H3 (nuclei).

### 4.22 DNA Copy Number

DNA was isolated using a Qiagen DNA isolation kit (69506). DNA copy number was determined by qPCR from DNA isolated from the total and mitochondrial fractions. Mitochondrial and nuclear genes were quantified by qPCR, and DNA levels were expressed as relative to *Rbs18* in the total DNA fraction.

### 4.23 Mitochondrial DNA Depletion

To induce mitochondrial DNA (mtDNA) depletion, NIH3T3 cells cultured in DMEM supplemented with 10% fetal bovine serum (FBS) and 1% penicillin/streptomycin (P/S) were treated with 100 ng mL^−1^ ethidium bromide (EtBr) and 50 μg mL^−1^ uridine (U3003‐56). Starting at 20 days of treatment, total DNA was extracted, and mtDNA levels were quantified by qPCR using the mitochondrial gene probes for Cox1, Cytb, Nd1, and Nd3. When mtDNA depletion was at 50% or higher, EtBr‐treated and control cells were cultured in either NAM or NAM Free media for an additional 7 days. RNA was then isolated for qPCR analysis.

### 4.24 Lipid Peroxidation Detection

Malondialdehyde (MDA), a marker of lipid peroxidation, was measured in cells (2 × 10^4^ cells) following the manufacturer's protocol (Abcam, 118970).

### 4.25 Catalase and Superoxide Dismutase Activity

NIH3T3 cells (1 × 10^6^ cells) cultured in NAM or NAM Free media were rinsed twice with HBSS and sonicated three times. The cell lysate was then centrifuged at 10,000 *g* for 10 min, and the supernatant was collected for enzymatic activity assays. Catalase (CAT) activity was assessed by measuring the decrease in absorbance at 240 nm over 1 min in 1 mL of HBSS containing 10 mM H_2_O_2_ (Pereira‐Maróstica et al. 2019). Superoxide dismutase (SOD) activity was determined based on its ability to inhibit pyrogallol autoxidation in a buffer containing 200 mM Tris base and 2 mM EDTA (pH 8.2), with absorbance measured at 420 nm (Pereira‐Maróstica et al. 2019). Enzyme activities were corrected by total protein concentration and expressed relative to cells cultured in NAM media.

### 4.26 Graphing and Statistical Analyses

Data are the mean ± SEM analyzed by an unpaired two‐sided *t*‐test or one‐way ANOVA as described in the figure legends. *p* < 0.05 was considered significant. GraphPad Prism software versions 7.04, 9.2.0, and 10.0.1 (GraphPad Software, San Diego, CA) were used for analysis.

## Author Contributions

C.C.S.C., E.N.C., and L.C. planned and conceptualized the study, analyzed data, and wrote the manuscript. C.C.S.C., L.C., E.P., and J.L.S. performed cell culture, biochemical assays, and analyzed data. S.K. and S.M. performed RNA‐seq analysis. B.H., A.B.‐R., S.P.R., G.H.S., and G.L.C.L. performed biochemical assays. J.V., R.G.M., and M.L.M.‐F. were involved in the preparation of the manuscript. All authors revised and approved the final version of the article.

## Conflicts of Interest

E.N.C. holds a patent on the use of CD38 inhibitors for metabolic diseases that is licensed by Elysium Health. E.N.C. is a consultant for TeneoBio, Calico, Mitobridge, and Cytokinetics. E.N.C. is on the advisory board of Eolo Pharma. E.N.C. owns stocks in TeneoBio. Research in the Chini laboratory has been conducted in compliance with the Mayo Clinic conflicts of interest policies. The other authors declare no competing interests.

## Supporting information


Figures S1–S7

Tables S1–S2

## Data Availability

The data that support the findings of this study are available from the corresponding author upon reasonable request.
