# Supplementary material for: Chronic Cellular NAD Depletion Activates a Viral Infection‐Like Interferon Response Through Mitochondrial DNA Leakage
Source: Aging Cell. 2025 Jun 16;24(9):e70135. doi: 10.1111/acel.70135 (PMC12419838; doi:10.1111/acel.70135)
Supplement: Supplementary file 1 — Figures S1–S7 [file ACEL-24-e70135-s001.pdf]

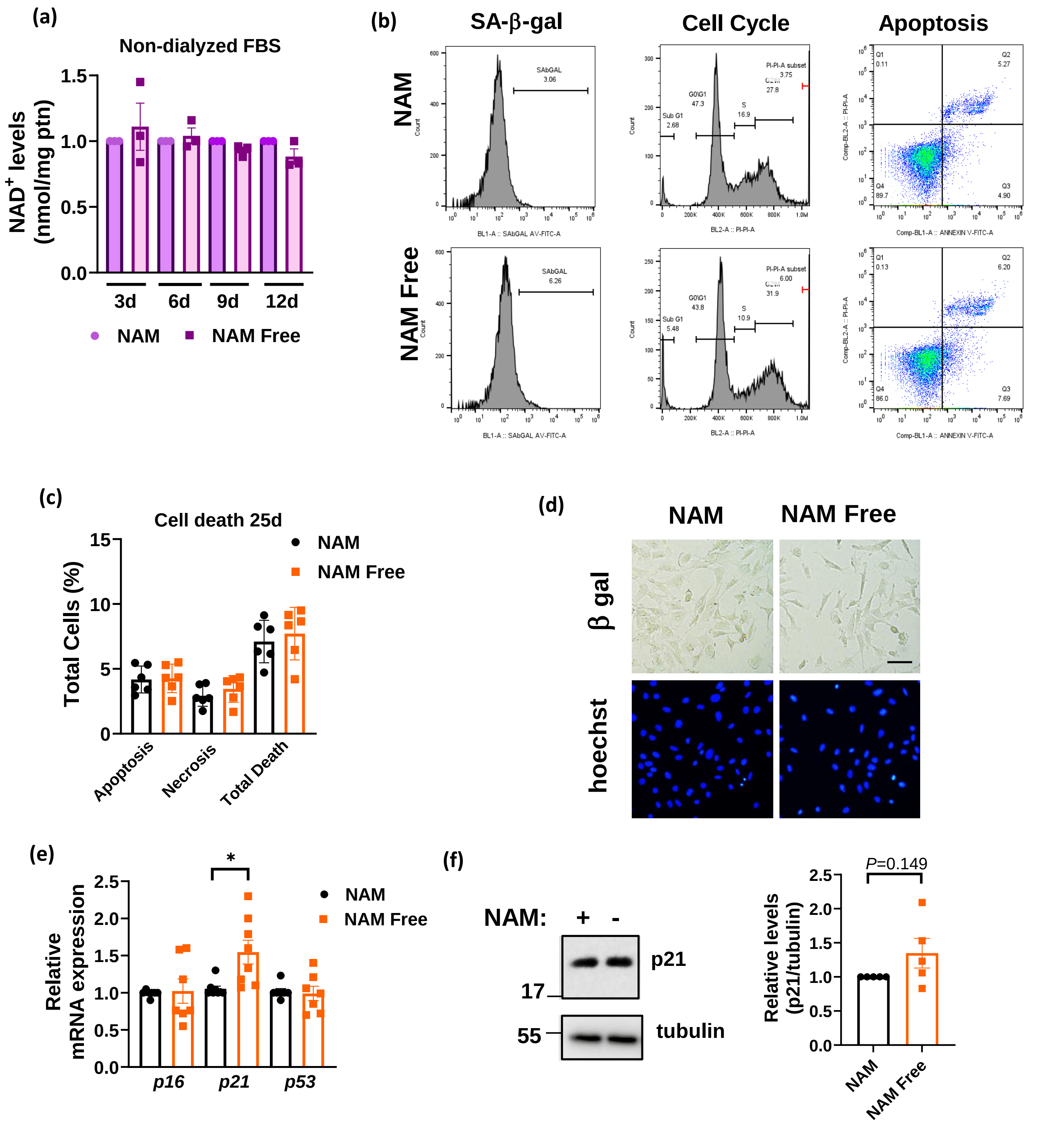

Supplementary Figure S1. Nicotinamide depletion impairs cell proliferation, without inducing apoptosis, cell cycle arrest, or senescence. (a) NIH3T3 cells were cultured in NAM and NAM Free media using non-dialyzed FBS. (b-f) NIH3T3 cells were cultured for 7 to 9 days in NAM and NAM Free media containing dialyzed FBS. (b) SA-b-ga**l,** cell cycle profile, and apoptosis were assessed by flow cytometry (n=3). (c) Cells were cultured for 25 days in NAM and NAM Free media. Cell death was assessed by flow cytometry (n=6). (d) Representative SA-β-ga**l** staining at day 9. (Scale bar 41.7μm). (e,f) Expression of cell cycle arrest genes assessed by qPCR (d, n=7-8) or immunoblot (e, n=5). Data are presented as mean ± s.e.m., with *n* representing the number of experiments. *P* values were calculated using unpaired two-sided t-tests.

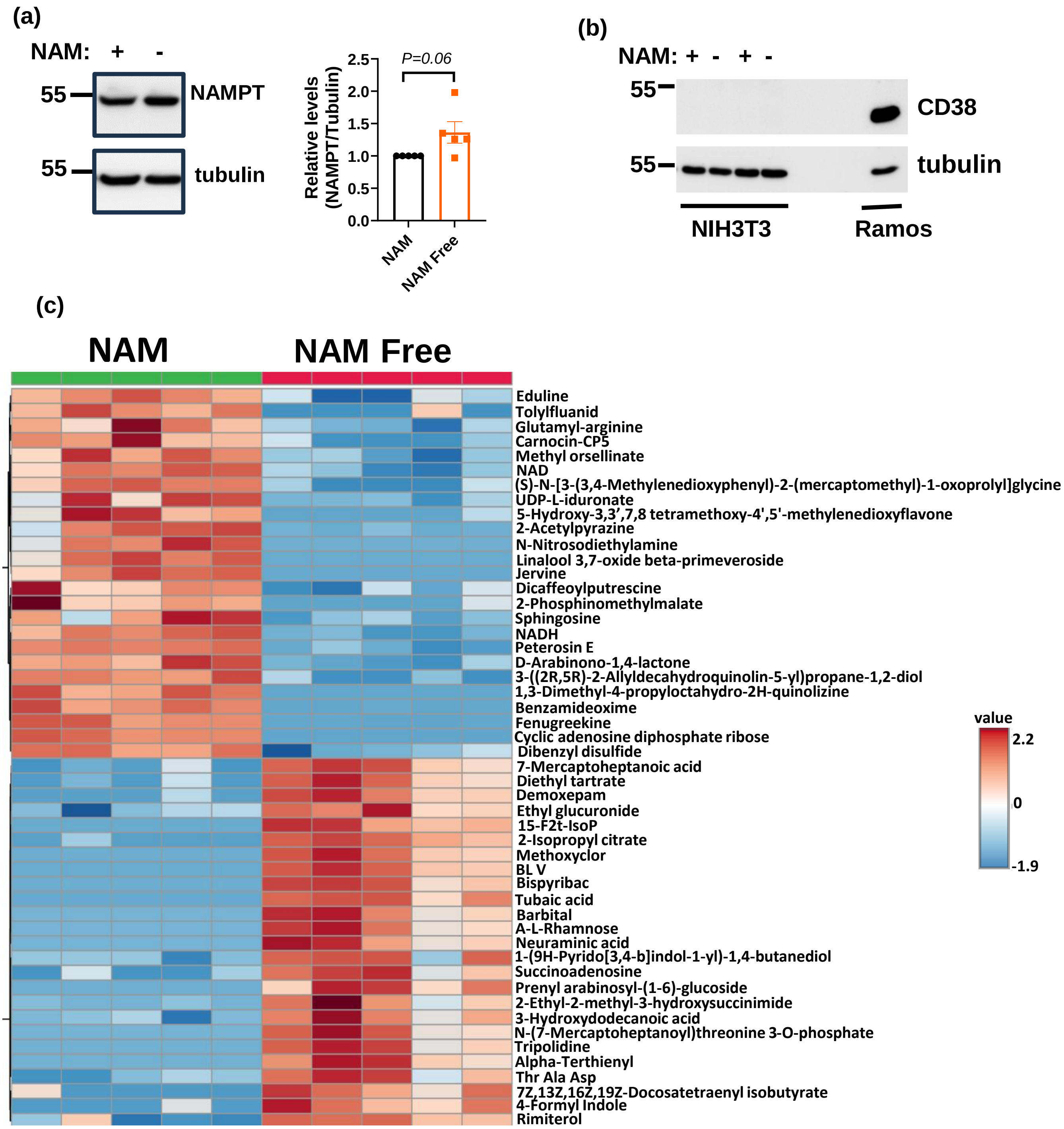

Supplementary Figure S2. Nicotinamide depletion promotes a metabolic shift. NIH3T3 cells were cultured for 9 days in NAM or NAM Free media. (a) Representative immunoblot of NAMPT levels. (b) Immunoblot of CD38 In NIH3T3 and Ramos cells. (c) Heat map shows the enrichment of the top 50 metabolites that were up and down regulated in NAM Free condition. Data analysis was performed using MetaboAnalyst 6.0 software (n=5).

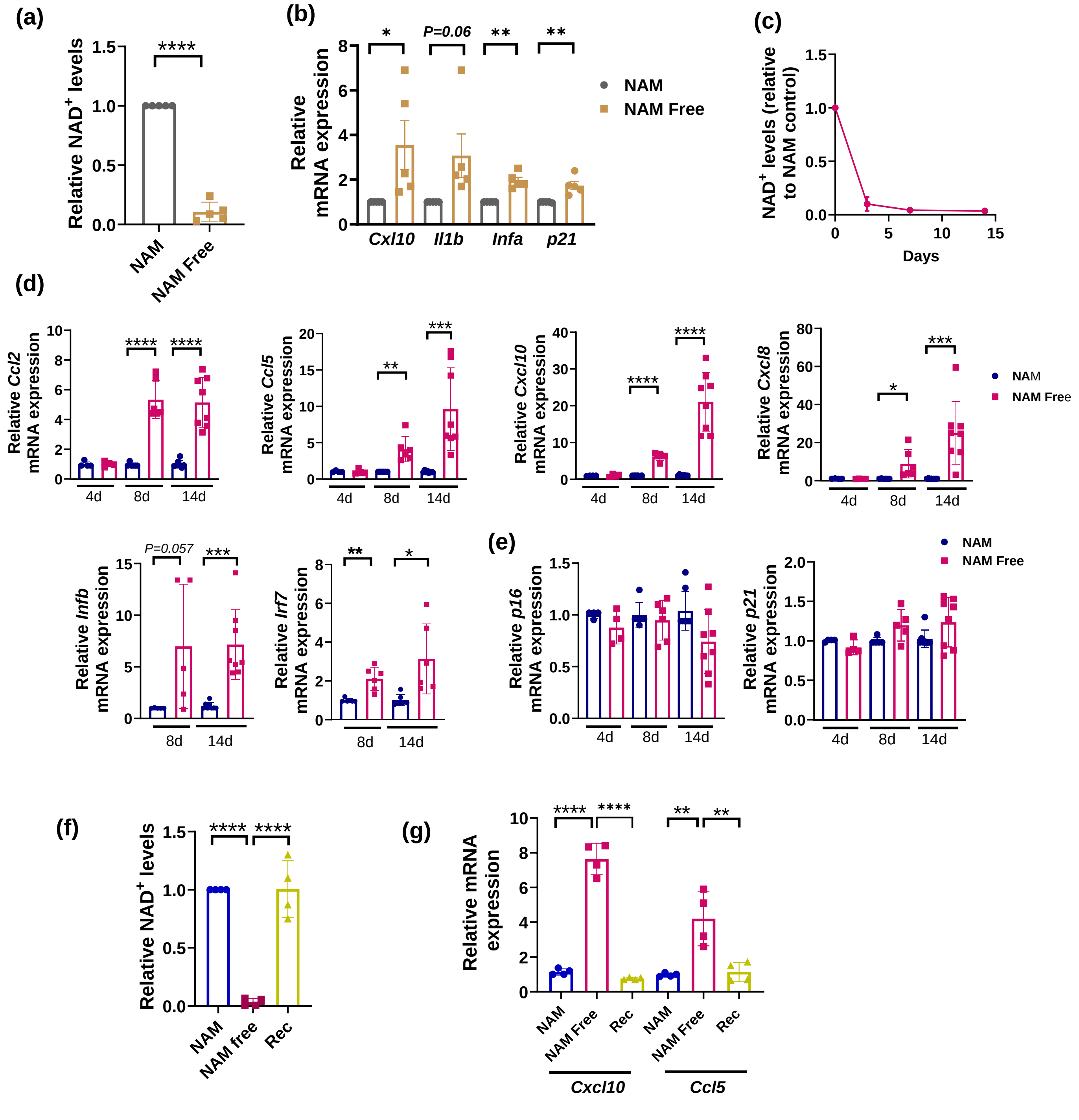

Supplementary Figure S3. Nicotinamide depletion regulates the inflammatory response in IMR90 human fibroblasts and HS5 cells. (a,b) IMR90 cells were cultured for 14 days in NAM or NAM Free media. (a) NAD levels relative to cells in NAM media. (b) Gene expression was quantified by qPCR (n=5). (c-e) HS5 cells were cultured in NAM or NAM Free media for varying durations. (c) NAD^+ levels in NAM Free cells are expressed relative to cells in NAM media (n=3-4). (d,e) Expression of inflammatory and cell cycle arrest genes were quantified by qPCR (n=4-8). (f,g) HS5 cells were cultured for 14 days in NAM, NAM Free, or NAM Free for 9 days followed by 5 days of recovery in NAM media (Recovery, Rec). (f) NAD^+ levels are expressed as relative to cells in NAM media. (g) Expression of inflammatory genes was quantified by qPCR (n=4). Data are presented as mean ± s.e.m., with *n* representing the number of experiments. *P* values were calculated using unpaired two-sided t-tests or one-way ANOVA.

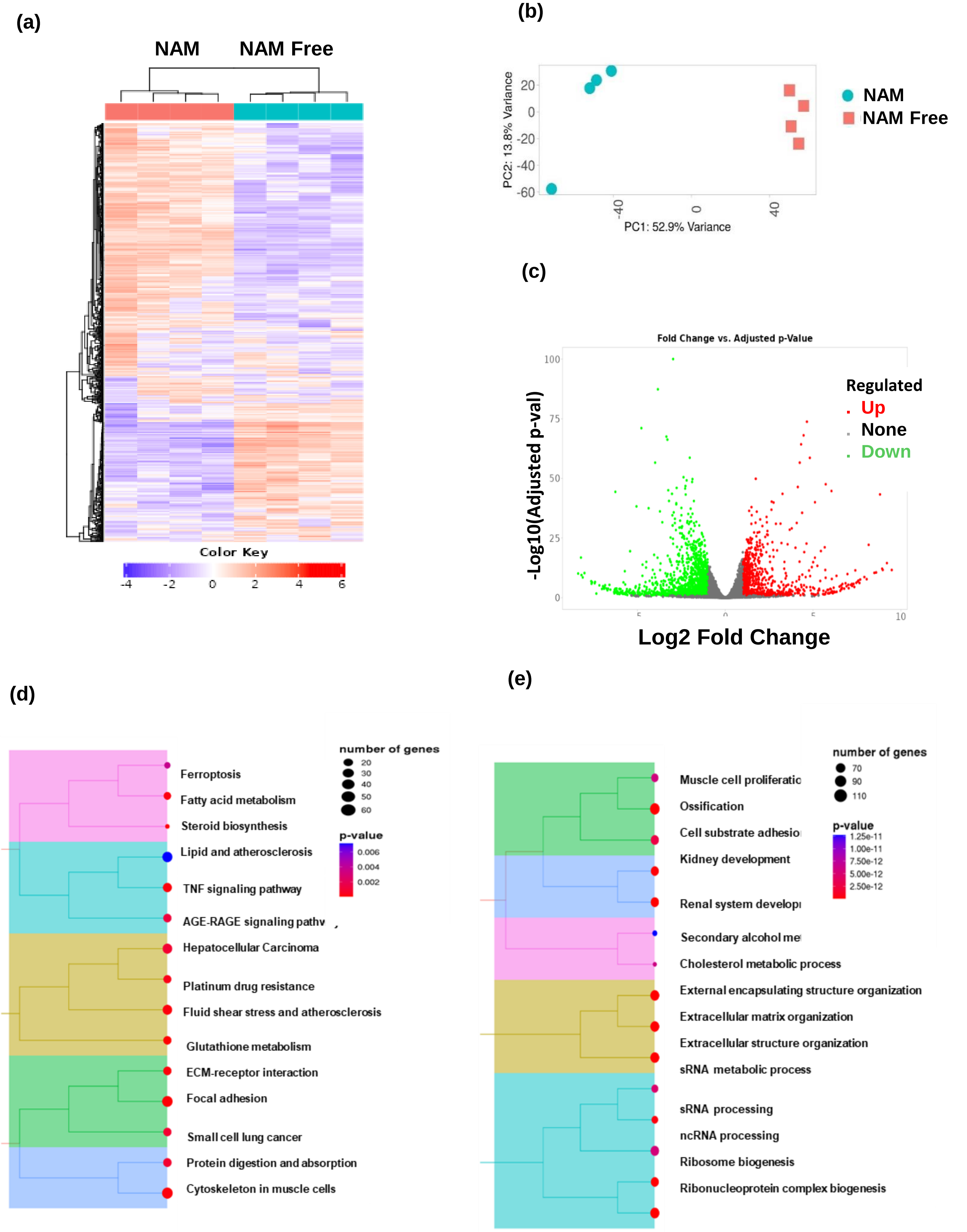
(a)
NAM
NAM Free
Color Key
-4
-2
0
2
4
6
(b)
PC2: 13.8% Variance
PC1: 52.9% Variance
20
0
-20
-40
-60
-40
0
40
NAM
NAM Free
(c)
Fold Change vs. Adjusted p-Value
-Log10(Adjusted p-val)
Log2 Fold Change
100
75
50
25
0
10
Regulated
Up
None
Down
(d)
number of genes
20
30
40
50
60
p-value
0.006
0.004
0.002
Ferroptosis
Fatty acid metabolism
Steroid biosynthesis
Lipid and atherosclerosis
TNF signaling pathway
AGE-RAGE signaling pathv
Hepatocellular Carcinoma
Platinum drug resistance
Fluid shear stress and atherosclerosis
Glutathione metabolism
ECM-receptor interaction
Focal adhesion
Small cell lung cancer
Protein digestion and absorption
Cytoskeleton in muscle cells
(e)
number of genes
70
90
110
p-value
1.25e-11
1.00e-11
7.50e-12
5.00e-12
2.50e-12
Muscle cell proliferatio
Ossification
Cell substrate adhesio
Kidney development
Renal system developi
Secondary alcohol me
Cholesterol metabolic process
External encapsulating structure organization
Extracellular matrix organization
Extracellular structure organization
sRNA metabolic process
sRNA processing
ncRNA processing
Ribosome biogenesis
Ribonucleoprotein complex biogenesis

Supplementary Figure S4. Main transcriptional pathways affected by NAM depletion in NIH3T3 cells. Cells were cultured for 12 days in NAM or NAM Free media (n=4). (a) Hierarchical clustering of the top 1000 genes affected by NAM depletion. (b) PCA analysis comparing NAM and NAM Free conditions. (c) Volcano plot highlighting the upregulated and downregulated DEGs induced by NAM depletion. (d,e) Enrichment tree plots illustrate overrepresented KEGG pathways (d) and Gene Ontology (GO) analysis (e).

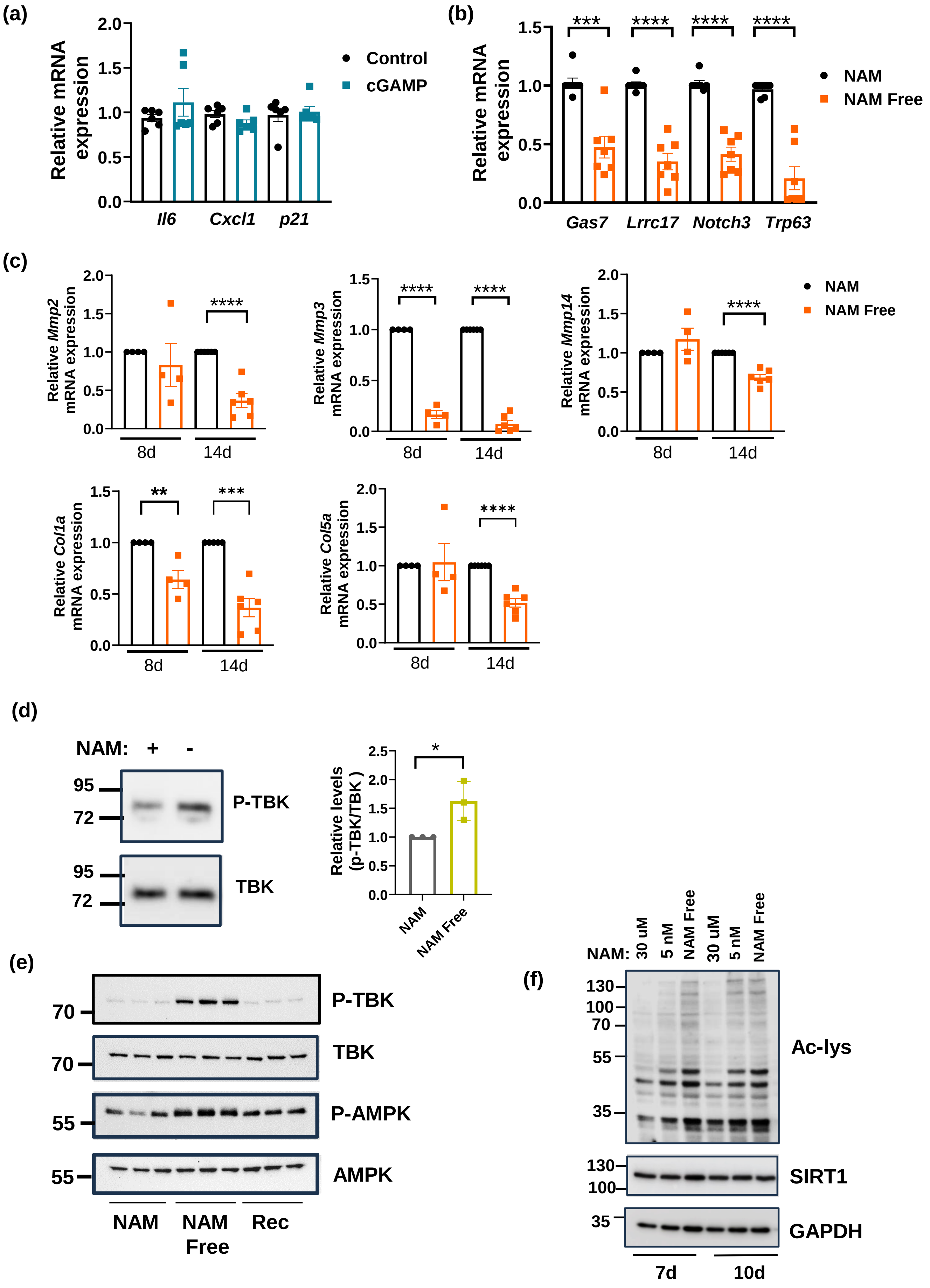
(a)
Relative mRNA expression
2.0
1.5
1.0
0.5
0.0
Il6
Cxcl1
p21
Control
cGAMP
(b)
Relative mRNA expression
1.5
1.0
0.5
0.0
***
****
****
****
Gas7
Lrrc17
Notch3
Trp63
NAM
NAM Free
(c)
Relative Mmp2 mRNA expression
Relative Mmp3 mRNA expression
Relative Mmp14 mRNA expression
Relative Col1a mRNA expression
Relative Col5a mRNA expression
8d
14d
****
**
***
NAM
NAM Free
(d)
NAM: + -
95
72
P-TBK
TBK
Relative levels (p-TBK/TBK)
2.5
2.0
1.5
1.0
0.5
0.0
*
NAM
NAM Free
(e)
70
55
P-TBK
TBK
P-AMPK
AMPK
NAM
NAM Free
Rec
(f)
NAM: 30 uM 5 nM NAM Free 30 uM 5 nM NAM Free
130
100
70
55
35
Ac-lys
SIRT1
GAPDH
7d
10d

Supplementary Figure S5. Gene expression and signaling pathways regulated by NAM depletion in NIH3T3, IMR90, and HS5 cells. (a) qPCR analysis of NIH3T3 cells treated with 15 μM cGAMP for 16 hours (n=5-6). (b,c) qPCR analysis of NIH3T3 cells cultured for 7 days (b) or 8 and 14d (c) in NAM or NAM Free media (n=4-7). (d) IMR90 cells were cultured for 14 days in NAM or NAM Free media. A representative immunoblot shows the levels of p-TBK and graph shows quantification relative to NAM conditions (n=3). (e,f) HS5 were grown in NAM and NAM Free media (7-22 days). In e, cells were grown for 22 days in NAM, NAM Free, or NAM Free for 15 days followed by 7 days of recovery in NAM media (Recovery, Rec.). Immunoblots show levels of phospho and total TBK and AMPK (n=3). (f) Immunoblot shows total acetylation and SIRT1 protein levels at 7 and 10 days. Immunoblot also show cells grown in 5 nM NAM. Data are presented as mean ± s.e.m., with *n* representing the number of experiments. *P* values were calculated using unpaired two-sided t-tests.

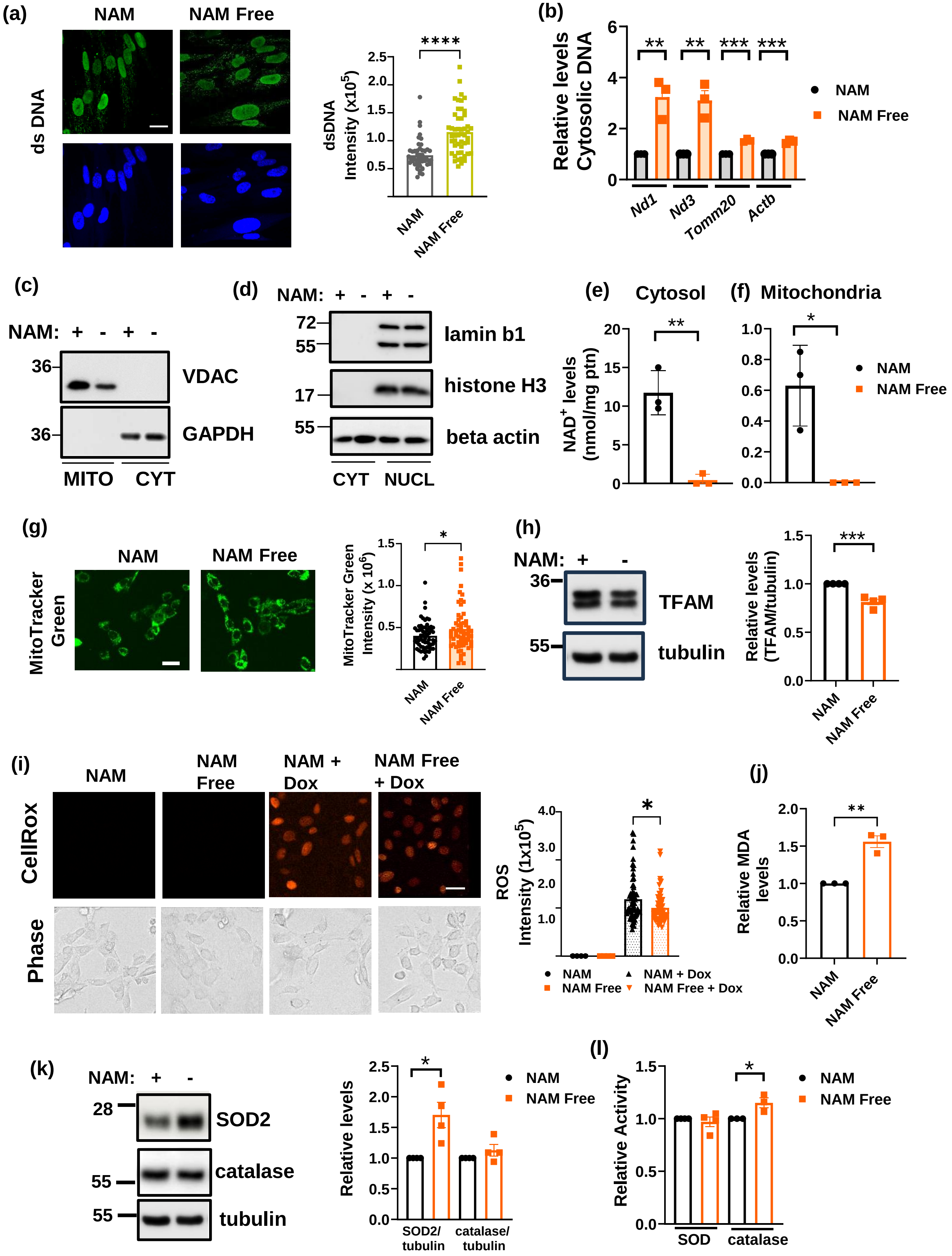

(a)
NAM
NAM Free
ds DNA
dsDNA Intensity (x10^5)
2.5
2.0
1.5
1.0
0.5
****
NAM
NAM Free
(b)
Relative levels Cytosolic DNA
6
4
2
0
**
**
***
***
NAM
NAM Free
Nd1
Nd3
Tomm20
Actb
(c)
NAM: + - + -
36
VDAC
36
GAPDH
MITO
CYT
(d)
NAM: + - + -
72
55
lamin b1
17
histone H3
55
beta actin
CYT
NUCL
(e)
Cytosol
NAD+ levels (nmol/mg ptn)
20
15
10
5
0
**
(f)
Mitochondria
1.0
0.8
0.6
0.4
0.2
0.0
*
NAM
NAM Free
(g)
NAM
NAM Free
MitoTracker Green
MitoTracker Green Intensity (x 10^6)
1.5
1.0
0.5
*
NAM
NAM Free
(h)
NAM: + -
36
TFAM
55
tubulin
Relative levels (TFAM/tubulin)
1.5
1.0
0.5
0.0
***
NAM
NAM Free
(i)
NAM
NAM Free
NAM + Dox
NAM Free + Dox
CellRox
Phase
ROS Intensity (1x10^5)
4.0
3.0
2.0
1.0
*
NAM
NAM Free
NAM + Dox
NAM Free + Dox
(j)
Relative MDA levels
2.0
1.5
1.0
0.5
0.0
**
NAM
NAM Free
(k)
NAM: + -
28
SOD2
55
catalase
55
tubulin
Relative levels
2.5
2.0
1.5
1.0
0.5
0.0
*
NAM
NAM Free
SOD2/ tubulin
catalase/ tubulin
(l)
Relative Activity
1.5
1.0
0.5
0.0
*
NAM
NAM Free
SOD
catalase

Supplementary Figure S6. Nicotinamide depletion regulates mitochondria and oxidative stress response. (a) IMR90 cells were cultured for 14 days in NAM or NAM Free media. Immunofluorescence shows the presence of cytosolic dsDNA and graph shows quantification from a representative experiment (scale bar 10µm). (b-k) NIH3T3 cells were cultured for 9 days in media with or without NAM. (b) After subcellular fractionation, the cytosolic fraction was used to assess presence of cytosolic DNA by qPCR (n=3). (c,d) Immunoblots of subcellular fractionation confirms the purity of the cytosolic (CYT) (c,d), mitochondrial (MITO) (c), and nuclear fractions (NUCL) (d). (e,f) NAD^+ levels in the cytosolic (e) and mitochondrial (f) fractions (n=3). (g) Fluorescence staining and quantification of a representative experiment with the mitochondrial content marker MitoTracker green (scale bar 31.2 µm). (h) Representative immunoblots show levels TFAM. Graph shows quantification (n=4). (i) Representative image and graph show CellRox staining. 0.5 µM doxorubicin (Dox) was added for the last 24 hours as a positive control (scale bar 15µm., n=3). (j) Lipid peroxidation assay measured the Malondialdehyde (MDA) marker levels and was expressed as relative to those in NAM treated cells (n=3). (k) Representative immunoblot and graph showing levels of SOD2 and catalase expressed as relative to NAM conditions (n=4). (l) SOD and catalase enzymatic activities (n=3-4). Data are presented as mean ± s.e.m., with *n* representing the number of experiments. *P* values were calculated using unpaired two-sided t-tests.

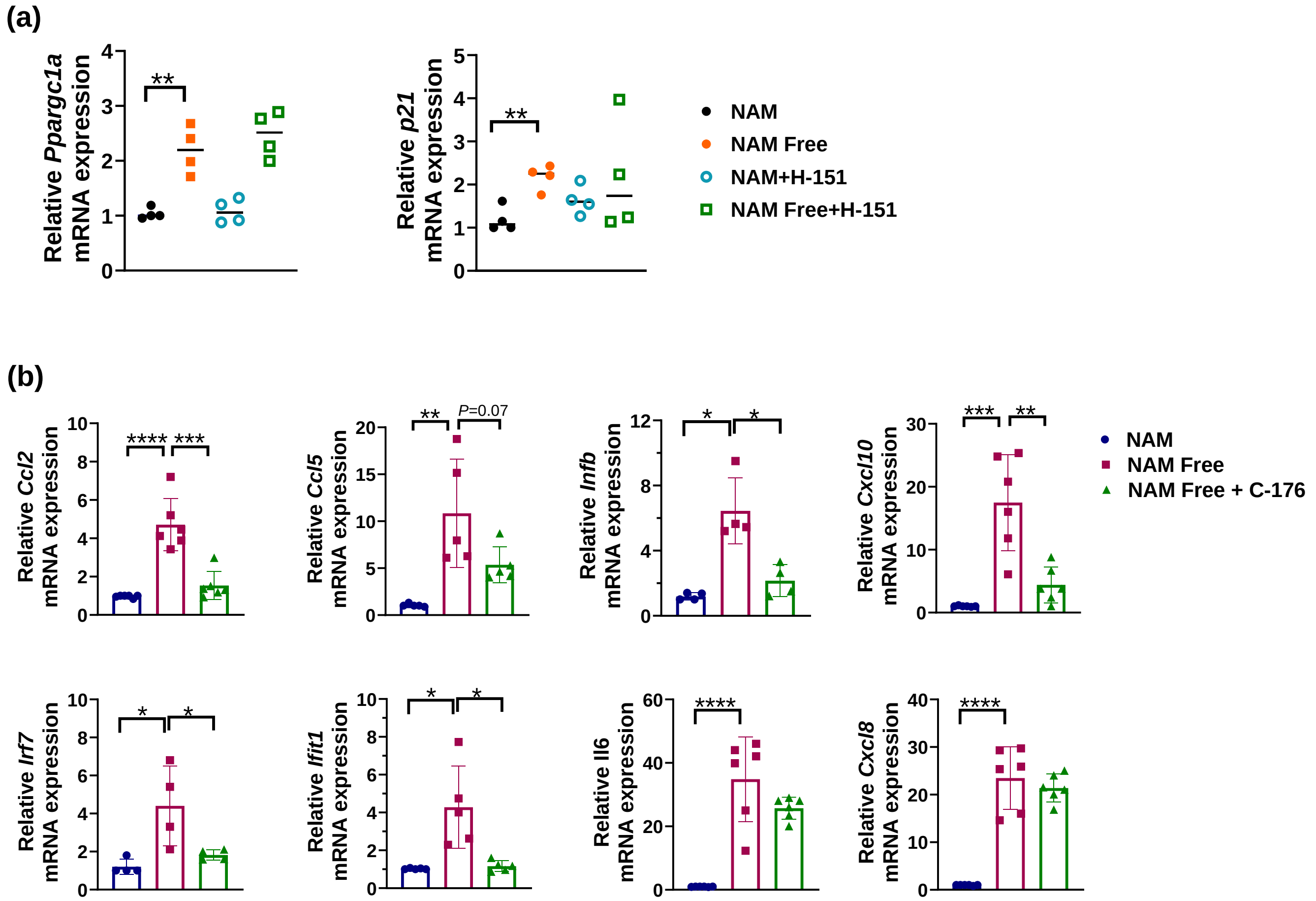

Supplementary Figure S7. Effect of STING inhibitors in the inflammatory response induced by NAM depletion. (a) qPCR analysis of NIH3T3 cultured for 7 days in NAM or NAM Free media. For the last 24 hours, cells were treated in with 0.5 µM H-151 (STING inhibitor) (n=4). (b) qPCR analysis of HS5 cells grown for 14 days in NAM or NAM Free media. For the last 24 hours, NAM Free cells were treated with or without 3 µM C-176 (STING inhibitor) (n=4-6). Data are presented as mean ± s.e.m., with *n* representing the number of experiments. *P* values were calculated using one-way ANOVA.
