# Supplementary material for: Chronic Cellular NAD Depletion Activates a Viral Infection‐Like Interferon Response Through Mitochondrial DNA Leakage
Source: Aging Cell. 2025 Jun 16;24(9):e70135. doi: 10.1111/acel.70135 (PMC12419838; doi:10.1111/acel.70135)
Supplement: Supplementary file 2 — Tables S1–S2 [file ACEL-24-e70135-s002.docx]

**Table 1. Antibodies**

**Dilution Cat # Company**

| Acetyl-lysine | 1/1000 | 9441 | Cell Signaling |
| --- | --- | --- | --- |
| AMPKα | 1/1000 | 2532 | Cell Signaling |
| AMPKα, Phospho (Thr172) (40H9) | 1/1000 | 2535 | Cell Signaling |
| GAPDH | 1/4000 | 97166S | Cell Signaling |
| IRF-3 | 1/1000 | 4302S | Cell Signaling |
| IRF-3, Phospho (S396) (D601M) | 1/1000 | 29047S | Cell Signaling |
| NAMPT | 1/1000 | 11776-1 | Proteintech |
| PAR/pADPr | 1/1000 | 4335 | R&D |
| p21 | 1/1000 | 37543 | Cell Signaling |
| p65 NFkB (L8F6) | 1/1000 | 6956 | Cell Signaling |
| p65 NFkB, Phospho (Ser 536) (93H1) | 1/1000 | 3033 | Cell Signaling |
| SIRT1 | 1/1000 | 2028 | Cell Signaling |
| SIRT3 | 1/1000 | 5490 | Cell Signaling |
| TBK1/NAK (D1B4) | 1/1000 | 3504S | Cell Signaling |
| TBK1/NAK, Phospho (S172) (D52C2) | 1/1000 | 5483S | Cell Signaling |
| Tubulin | 1/4000 | 15568 | Abcam |
| Mitofusin 2 | 1/1000 | 11925T | Cell Signaling |
| MFF | 1/1000 | 84580T | Cell Signaling |
| SOD 2 (Acetyl K68) (EPVANR2) | 1/1000 | 137037 | Abcam |
| SOD 2 | 1/1000 | 24127-1-AP | Protein Tek |
| Lamin B1 | 1/1000 | 216723 | Abcam |
| Histone H3 | 1/1000 | 9715 | Cell Signaling |
| p53 (Acetyl Lys379) | 1/1000 | 25705 | Cell Signaling |
| p53 | 1/1000 | 71818 | Santa Cruz |
| Beta-Actin (D6A8) | 1/4000 | 8457 | Cell Signaling |
| VDAC1 | 1/1000 | 4661S | Cell Signaling |
| TFAM | 1/1000 | PA5-29571 | Invitrogen |
| Catalase | 1/1000 | 14097 | Cell Signaling |

**Table 2 – Taqman probes**

| **Mouse** |  |
| --- | --- |
| *Ccl2* | Mm00441242_m1 |
| *Ccl5* | Mm01302427_m1 |
| *Ccl4* | Mm00443111_m1 |
| *Cdkn1a (P21)* | Mm04205640_g1 |
| *Cdkn2a (P16)* | Mm00494449_m1 |
| *Cd38* | Mm01220906_m1 |
| *Cd157* | Mm00477672_m1 |
| *Cxcl1* | Mm04207460_m1 |
| *Cxcl10* | Mm00445235_m1 |
| *Cytb* | Mm04225271_g1 |
| *Gas7* | Mm00433524_m1 |
| *Ifih1* | Mm00459183_m1 |
| *Ifit1* | Mm07295796_m1 |
| *Ifit3* | Mm01704846 |
| *Il6* | Mm00446190_m1 |
| *Irf7* | Mm00500226_m1 |
| *Lrrc17* | Mm01167263_m1 |
| *Mmp9* | Mm00442991_m1 |
| *Nampt* | Mm00451938_m1 |
| *Naprt1* | Mm01205844_g1 |
| *Nd3* | Mm04225292_g1 |
| *Nd4* | Mm04225294_s1 |
| *Nmnat1* | Mm01257929_m1 |
| *Nmnat2* | Mm00615393_m1 |
| *Nmnat3* | Mm00513791_m1 |
| *Notch3* | Mm01345646_m1 |
| *Parp1* | Mm01321084_m1 |
| *Parp14* | Mm00520984_m1 |
| *Ppargc1a* | Mm01208835_m1 |
| *TP53* | Mm01731290_g1 |
| *Sarm1* | Mm00555617_m1 |
| *Sirt1* | Mm00490758_m1 |
| *Sirt3* | Mm00452131_m1 |
| *Slc25a51* | Mm05911874_g1 |
| *Slc29a1(Ent1)* | Mm01270577_m1 |
| *Slc29a2(Ent2)* | Mm00432817_m1 |
| *Tbp* | Mm00446971_m1 |
| *Tomm20* | mm02601883_g1 |
| *Trp63* | Mm00495793_m1 |
| *18s* | Mm03928990_g1 |
| Mmp2 | Mm00439498_m1 |
| Mmp3 | Mm00440295_m1 |
| Mmp14 | Mm00485054_m1 |
| Col1a | Mm00801666_g1 |
| Col5a | Mm00489490_m1 |
| Nd1 | Mm04225274_s1 |
| Cox1 | Mm04225243_g1 |
| Actb | Mm02619580_s1 |

| **Human** |  |
| --- | --- |
| *Ccl12* | Hs00234140_m1 |
| *Ccl5* | Hs00982282_m1 |
| *Cxcl8* | Hs00174103_m1 |
| *Cxcl10* | Hs00171042_m1 |
| *Ifnb1* | Hs01077958_m1 |
| *Il6* | Hs00985639_m1 |
| *Irf7* | Hs01014809_g1 |
| *p16* | Hs00923894_m1 |
| *P21* | Hs00355782_m1 |
| *Tp53* | Hs01034249_m1 |
| *Ifna1* | Hs03044218_g1 |
| *Il1b* | Hs01555410_m1 |
